# Supplementary material for: CombiANT reader: Deep learning-based automatic image processing tool to robustly quantify antibiotic interactions
Source: PLOS Digit Health. 2025 Jul 8;4(7):e0000669. doi: 10.1371/journal.pdig.0000669 (PMC12237020; doi:10.1371/journal.pdig.0000669)
Supplement: S1 Appendix — A detailed explanation of the CombiANT assay and its annotation process. (PDF) [file pdig.0000669.s001.pdf]

CombiANT

The CombiANT assay is shown in Fig 1. Opposite each reservoir, at the outer side of the white circle mark, the antibiotic acts alone as the concentration of the others there is negligible. The inscribed white triangle mark constitutes the interaction area, where antibiotics act in pairs, with the highest combination concentration at the triangle vertices. Due to the distance to the opposite reservoir, the concentration of the third antibiotic is negligible at each triangle vertex. Antibiotics A and B act together in the bottom right, A and C in the bottom left, and B and C in the top of the triangle. The darker areas in the assay exhibit uninhibited bacterial growth. The assay has two growth zones: an "inner" inside the interaction zone triangle and an "outer" outside the circle. In the original CombiANT test, a human has to manually annotate key points on the edges of the growth zones and also pinpoint the triangle vertices: ICA, ICB, and ICC are inhibitory concentration points placed at the bacteria boundary opposite the midpoint of the corresponding reservoir on a straight line perpendicular to the circle perimeter. Three combination inhibitory points, CPAB, CPAC, and CPBC, are placed on the rim of the inner growth zone at the closest point to the respective triangle vertex. Finally, the evaluator has to annotate the vertices of the triangle mark interaction zone in the correct order: VAB, VAC, VBC, enabling the original CombiANT software to align all coordinates on the assay with the pre-calculated diffusion landscape. After this manual point-annotation, the required distances can be obtained, shown as dashed lines in Fig 1.

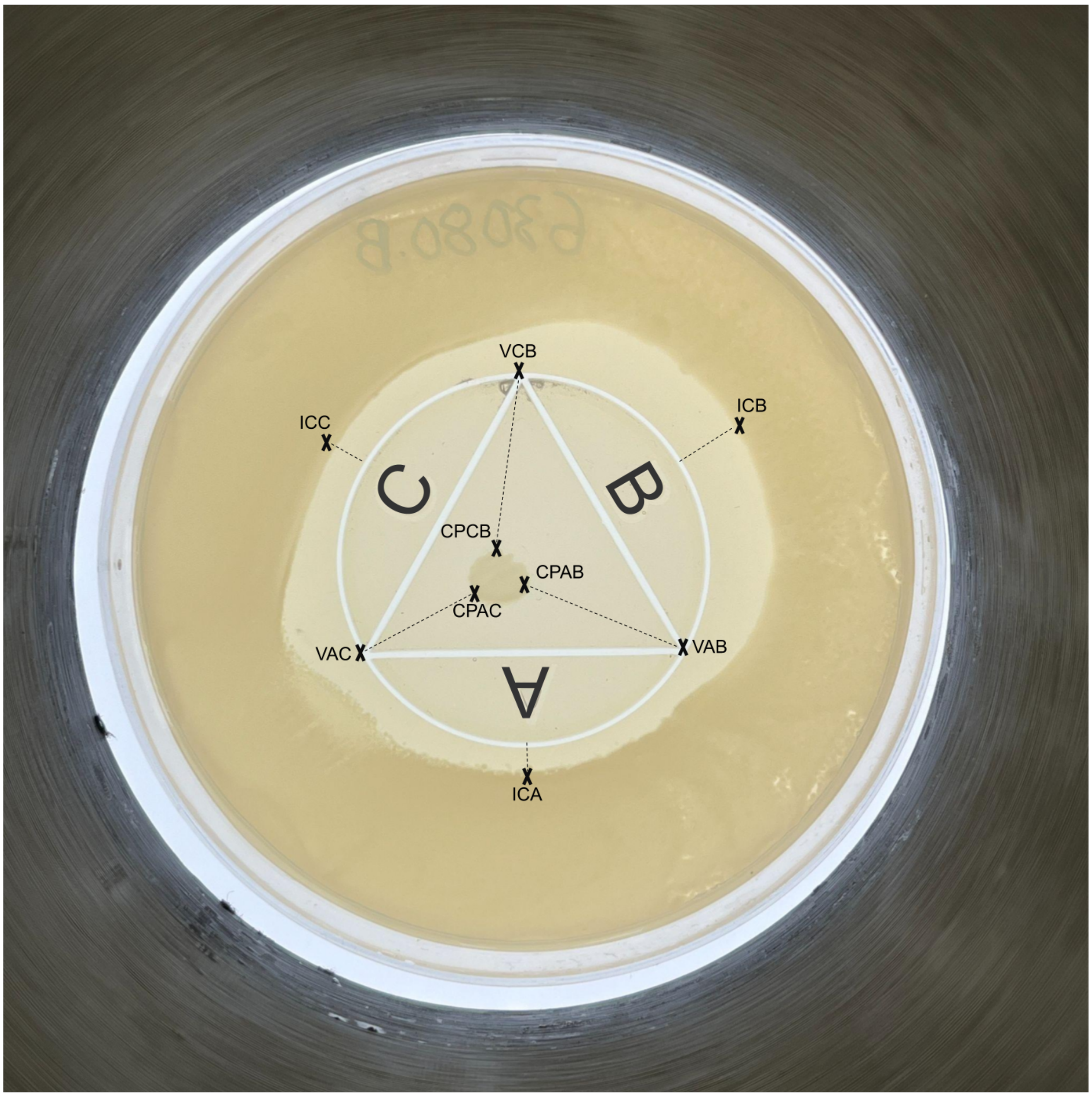

Figure 1: A CombiANT assay with nine annotated key points required to be manually pinpointed by a human evaluator. Dashed lines outline the obtained distances. ICA, ICB, and ICC are inhibitory concentration points that indicate the effects of the corresponding antibiotics acting alone. Due to irregularities of the outer growth zone boundary, this value can be inconsistent. CPAB, CPAC, and CPBC are combination inhibitory points, and VAC, VAB, and VCB are triangle vertices. The three antibiotic inserts (A, B, and C) are filled in black for visibility.
