# Supplementary material for: CombiANT reader: Deep learning-based automatic image processing tool to robustly quantify antibiotic interactions
Source: PLOS Digit Health. 2025 Jul 8;4(7):e0000669. doi: 10.1371/journal.pdig.0000669 (PMC12237020; doi:10.1371/journal.pdig.0000669)

Discards

In total, 7 plates were flagged for discarding by the software, shown in the following images. However, these plates were still retained in our evaluation due to containing useful distances.

Plate 10

The inner growth zone has grown out of the interaction area into well A.

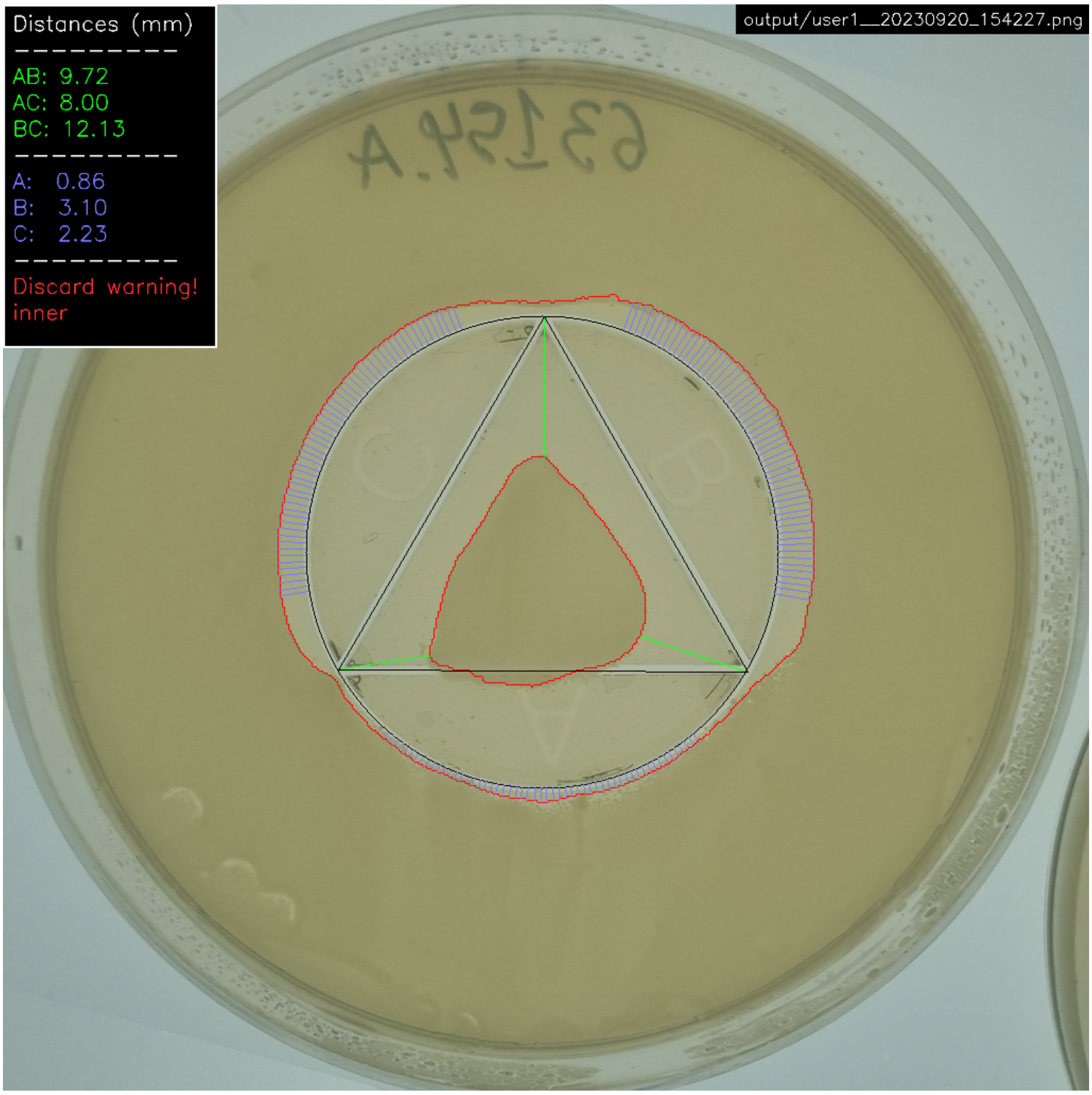

Plate 29

The inner growth zone has grown out of the interaction area into well A and connected with the outer growth zone.

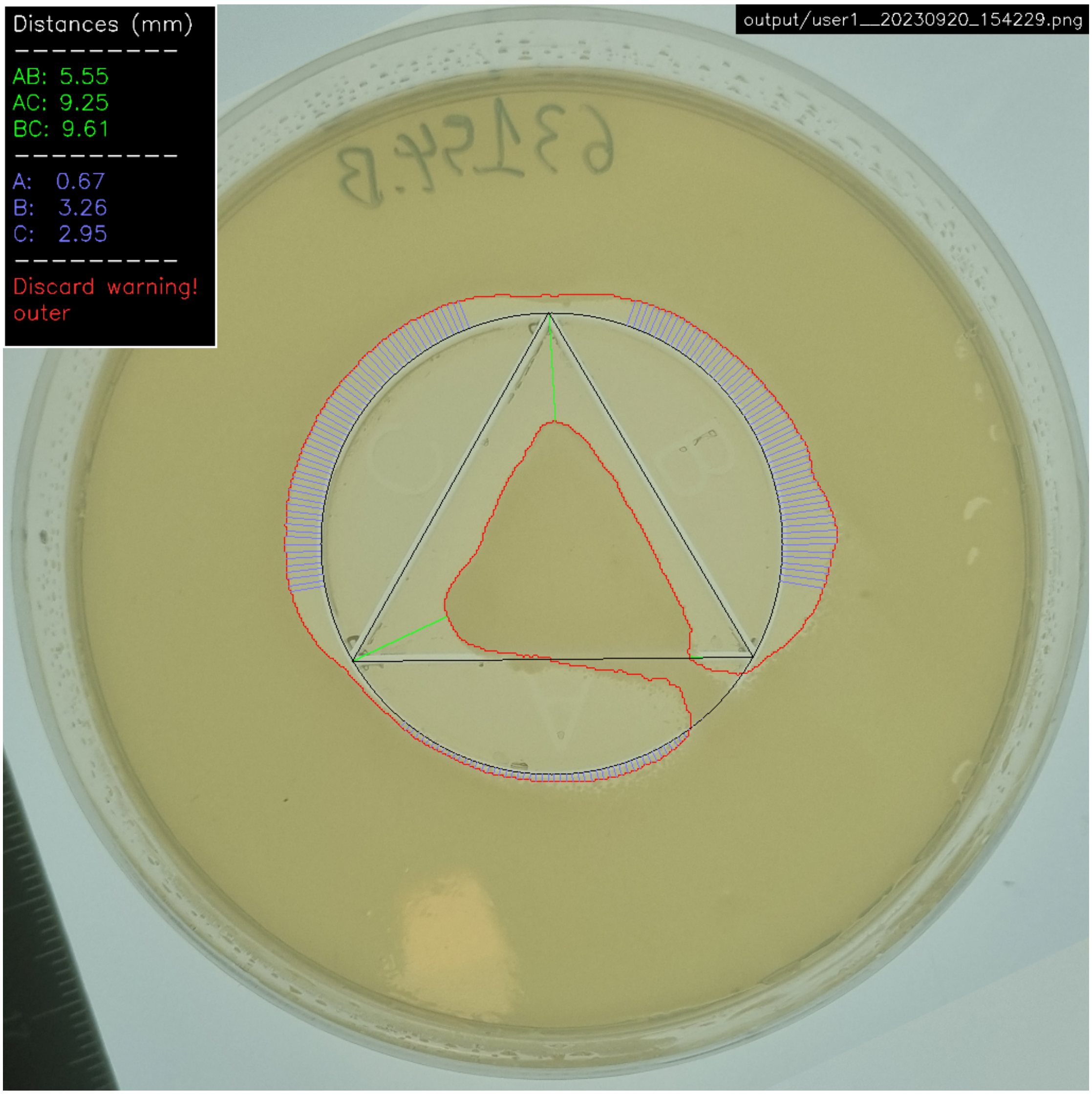

Plate 44

The outer growth zone has grown into well B. The bacterial growth covers the circle mark, causing a slight error in the template matching procedure.

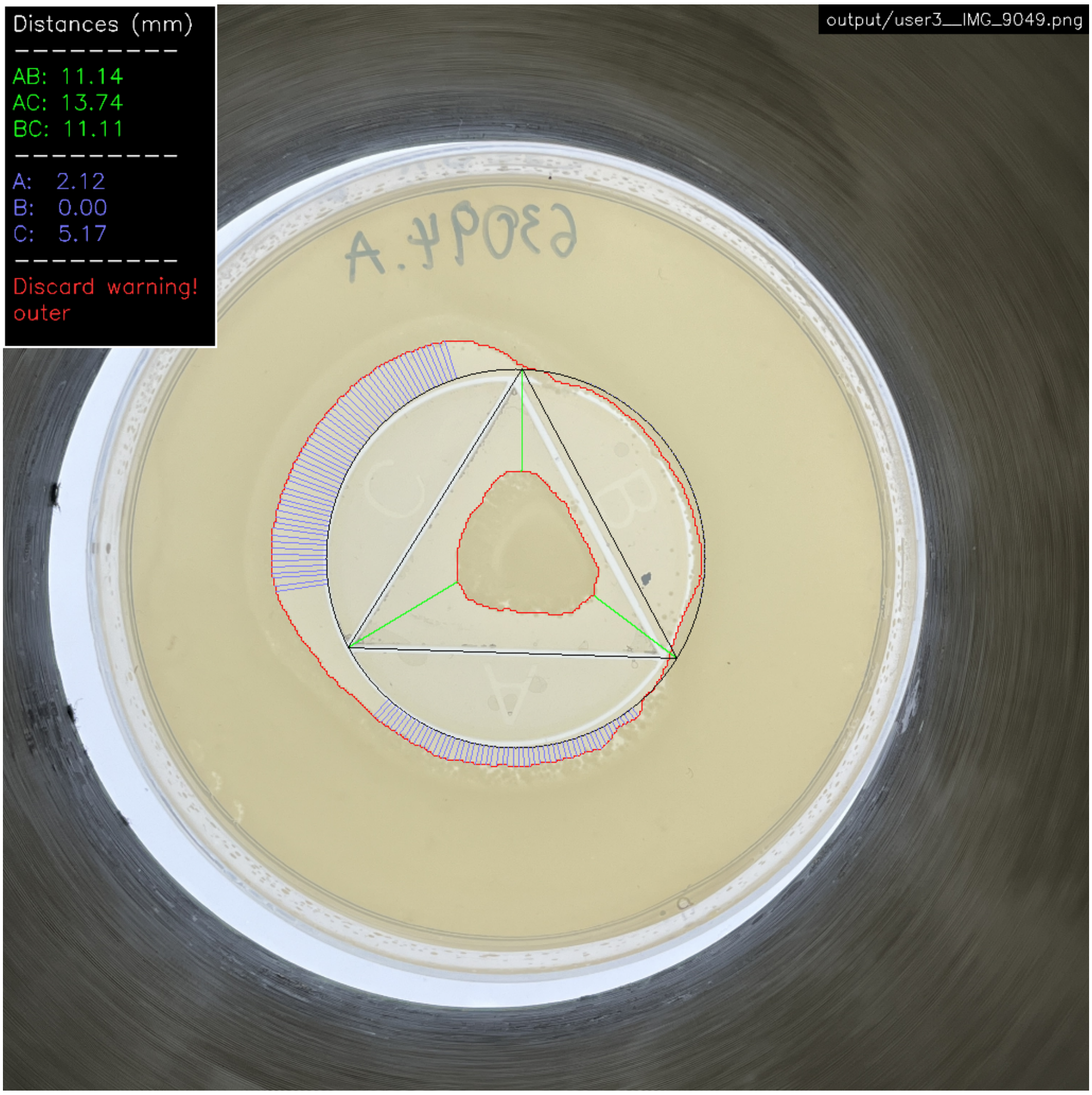

Plate 48

The outer growth zone has grown into well B.

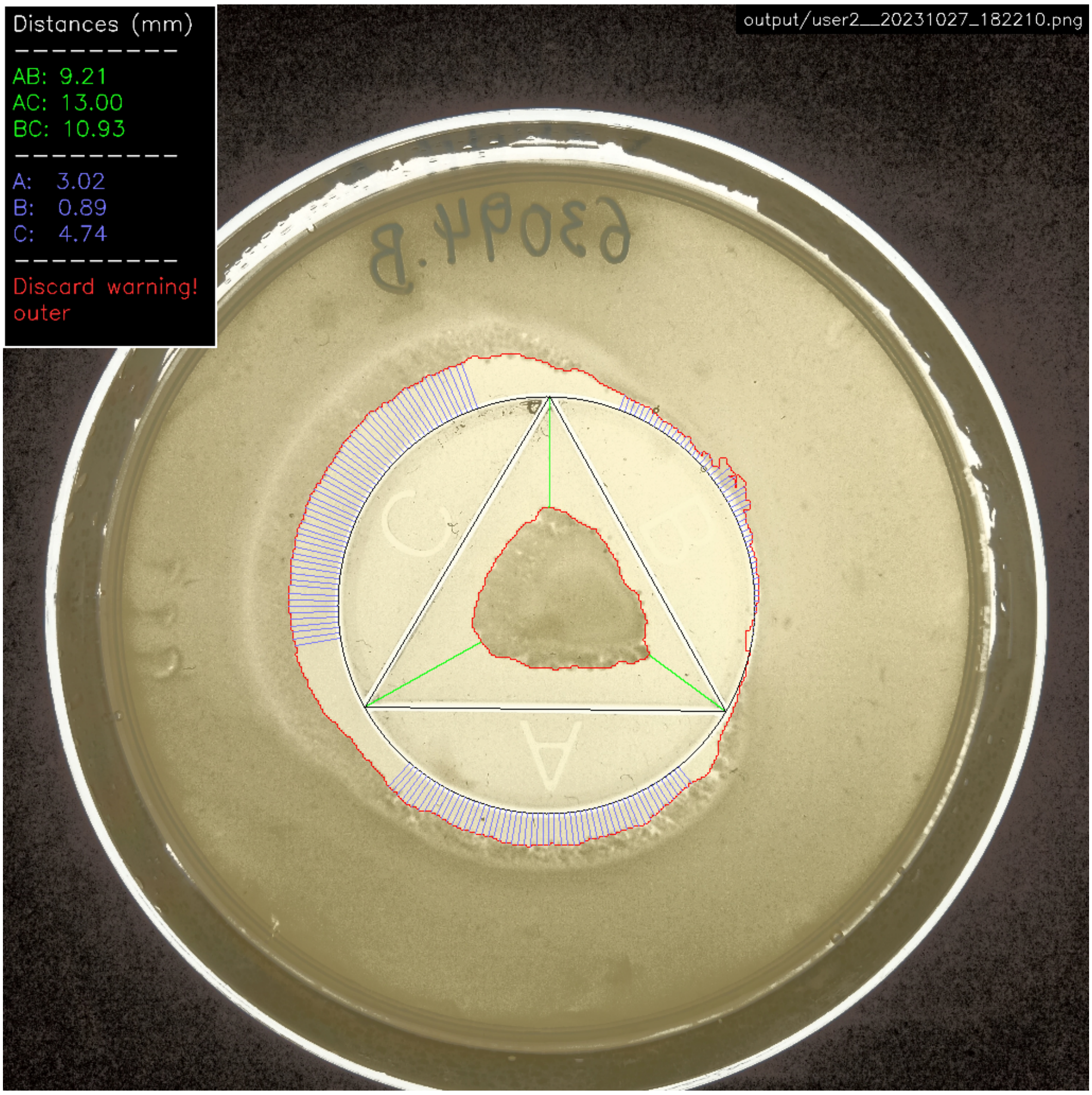

Plate 62

The outer growth zone has grown into well A.

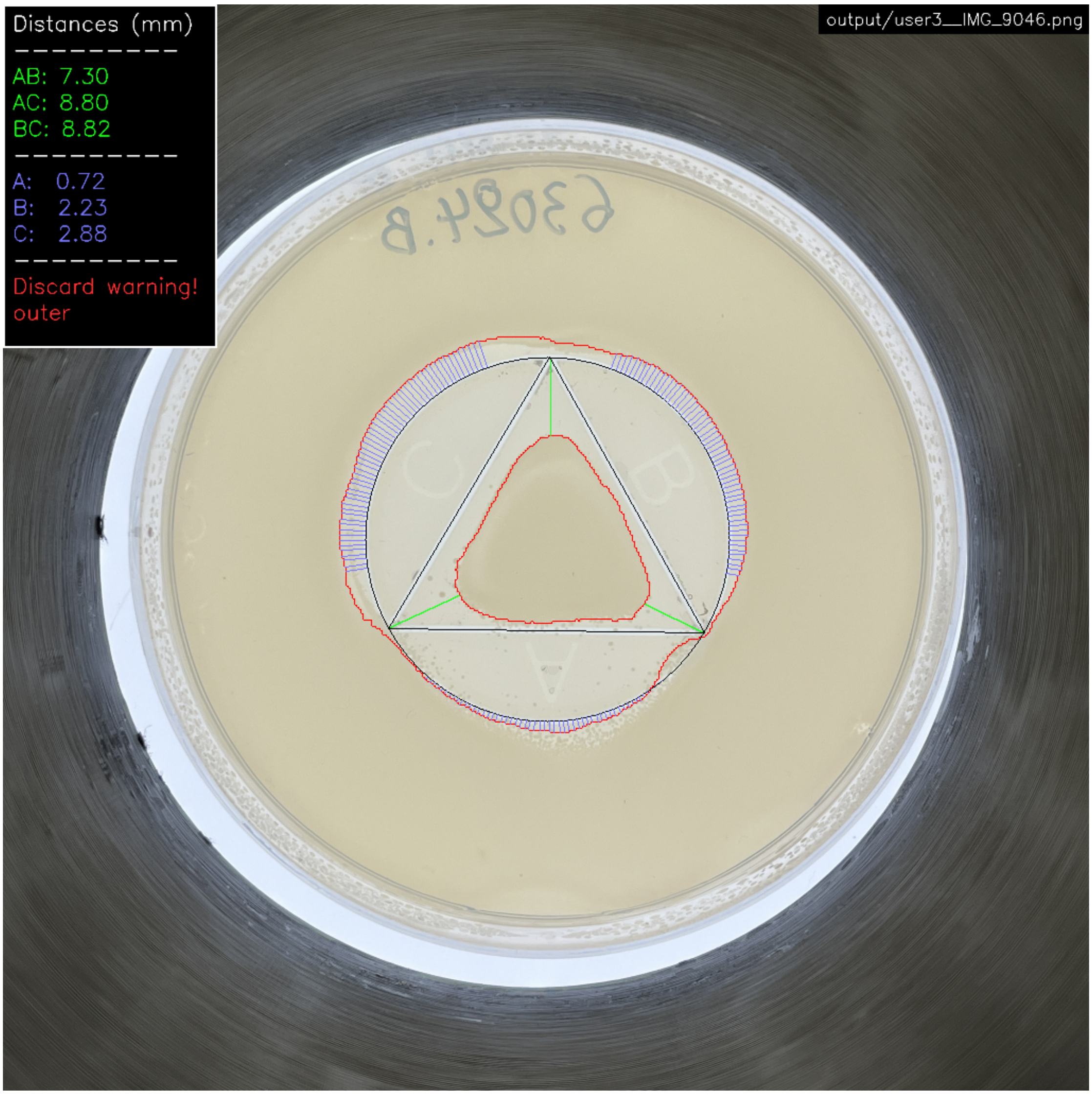

Plate 75

The inner growth zone has grown out of the interaction area into well A. Also the outer growth zone has grown into well A from the other side.

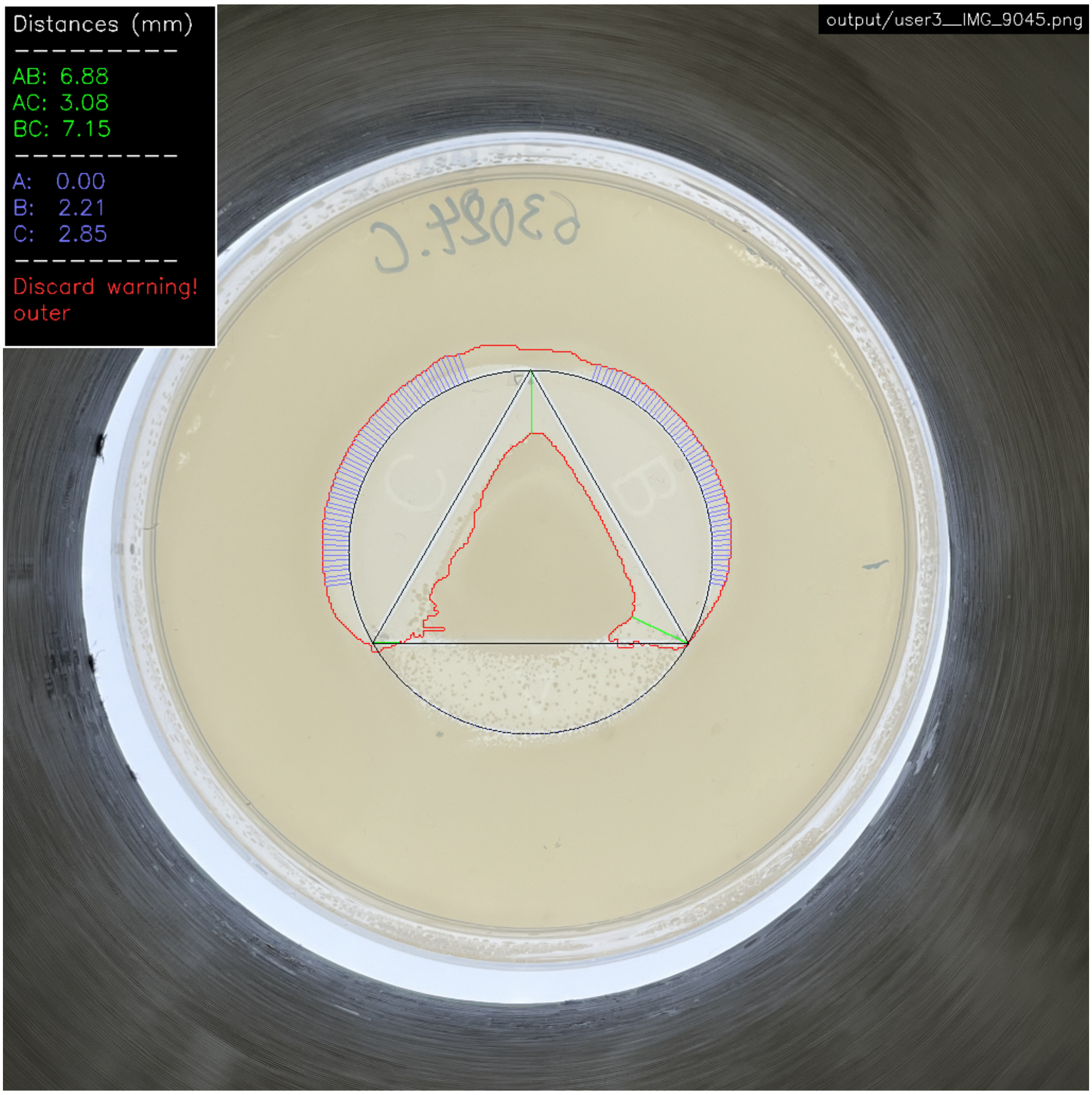

Plate 82

The outer growth zone has grown into well A.

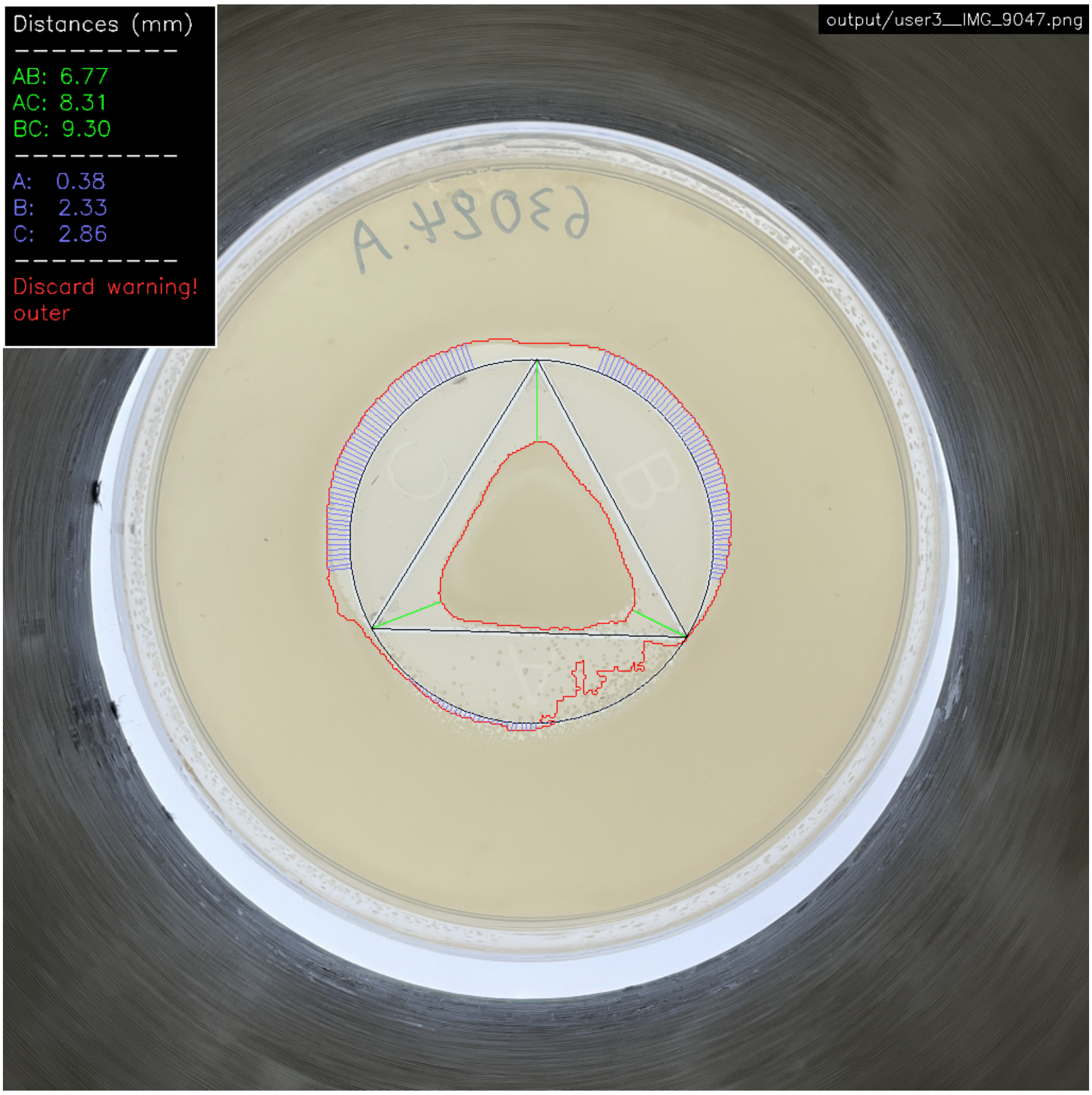

Supplement: S2 Appendix — All seven plates flagged for discarding are visualized in one document, along with the rationale for why the plates were discarded. (PDF) [file pdig.0000669.s002.pdf]
