# Supplementary material for: CombiANT reader: Deep learning-based automatic image processing tool to robustly quantify antibiotic interactions
Source: PLOS Digit Health. 2025 Jul 8;4(7):e0000669. doi: 10.1371/journal.pdig.0000669 (PMC12237020; doi:10.1371/journal.pdig.0000669)

Outliers

There were five absolute error measurements above 3.5 millimeters, originating from a total of four plates, shown in Table 1. In the following pages, each plate is shown along with the user and software measurements, outliers are marked with a blue cell color. The table is followed by the photograph of the plate containing the outlier.

Table 1: **Outliers** Distances with absolute errors above 3.5mm between the user and software gradings.

Name	Plate ID	User	Distance Name	Absolute error (mm)
IMG_9006	36	Beginner	B	3.605
IMG_9008	42	Beginner	B	6.482
20231027_182937	79	Experienced	AB	4.854
IMG_9005	4	Beginner	AB	4.842
IMG_9005	4	Beginner	AC	4.675

Outlier in plate 36

Clearly, the beginner user made a wrong assessment of the distance B.

Plate ID	User	B (software)	B (user)
36	Intermediate	5.567	4.701
36	Experienced	5.792	5.398
36	Beginner	5.657	2.052

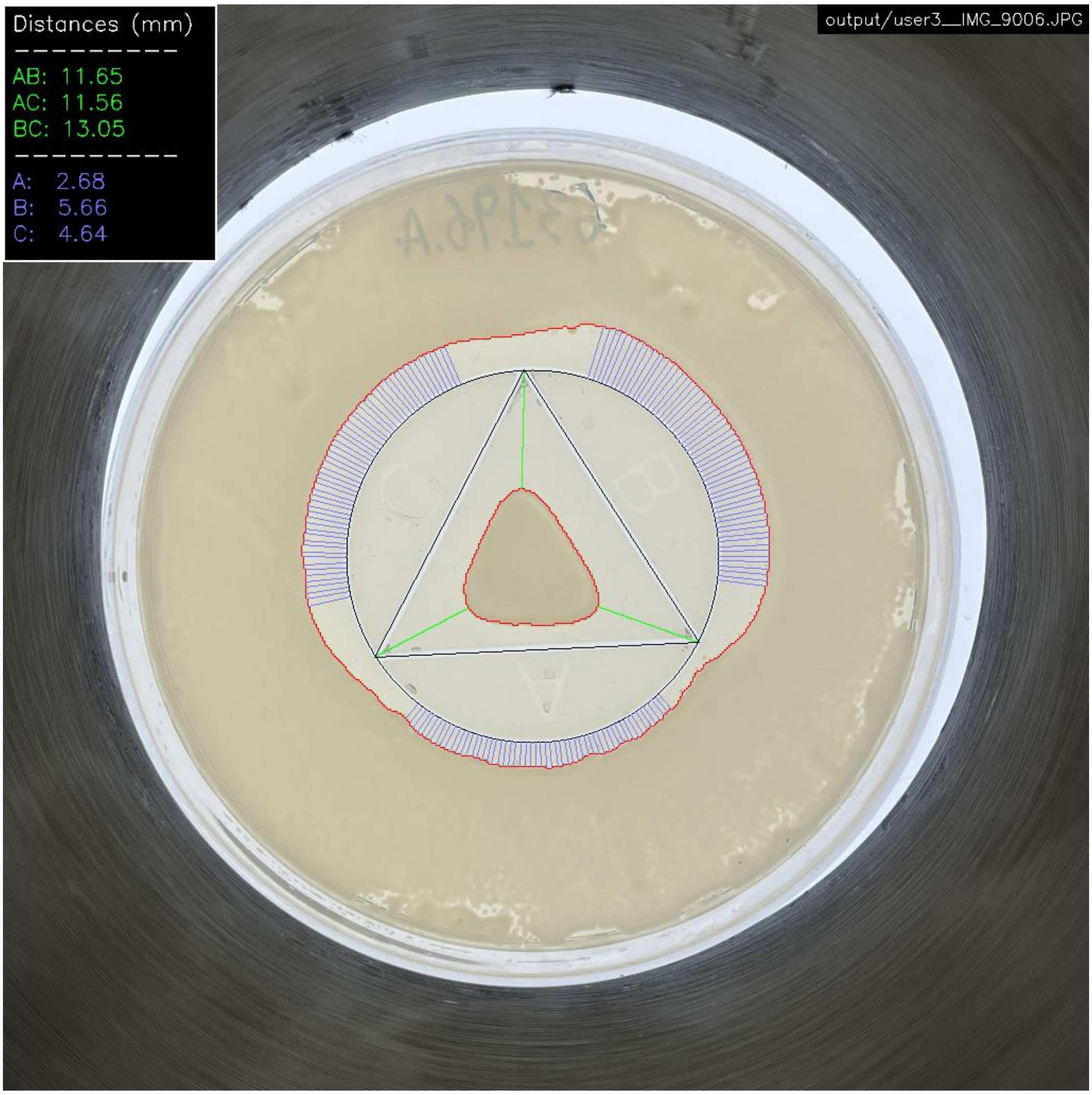

Outlier in plate 42

Clearly, the beginner user made a wrong assessment of the distance B.

Plate ID	User	B (software)	B (user)
42	Intermediate	1.749	1.652
42	Experienced	2.351	1.838
42	Beginner	1.882	8.363

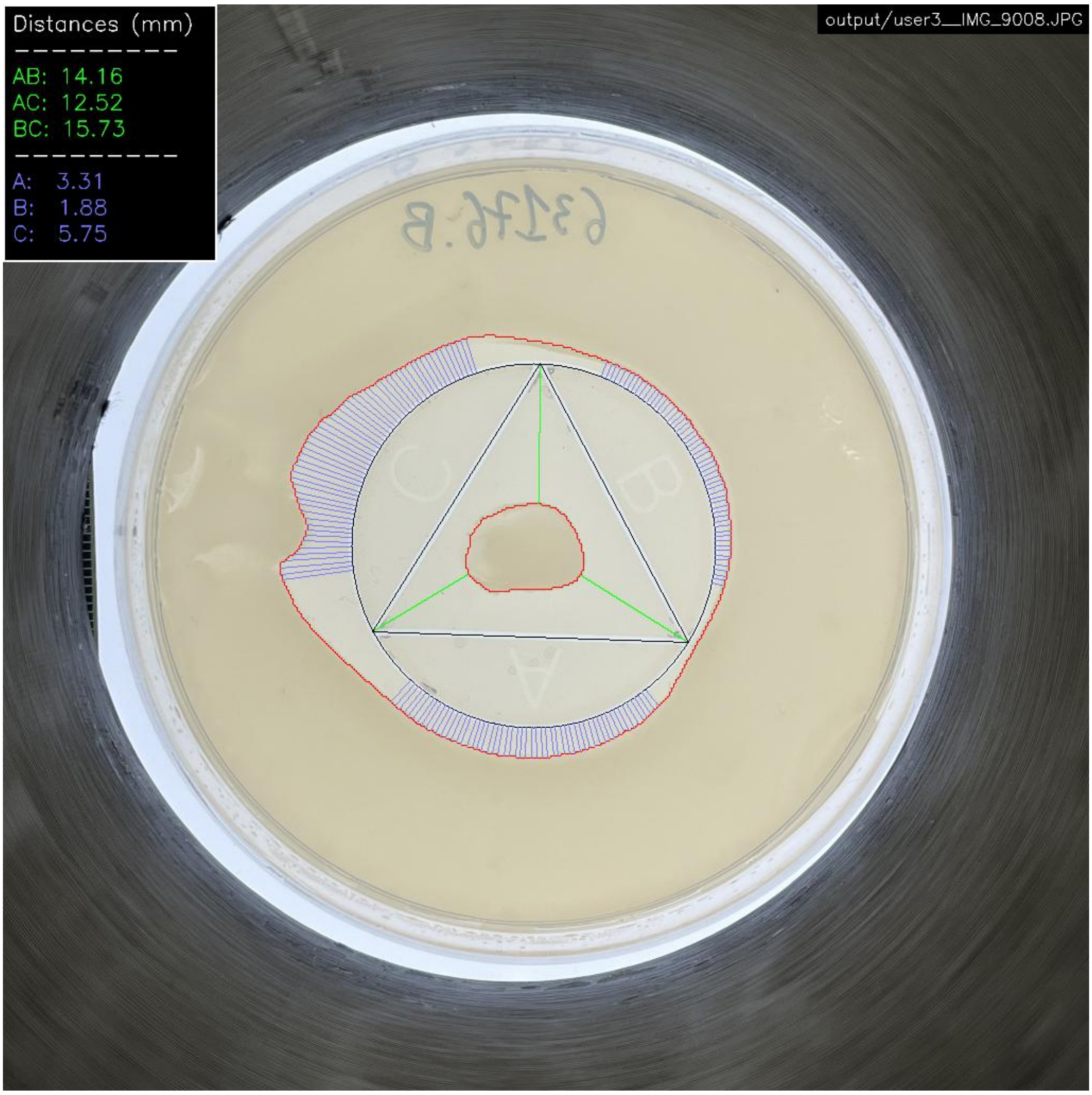

Outlier in plate 79

In this plate, the software made the wrong assessment in the photograph taken by the experienced user. The U-Net is picking up the dark spot as bacterial content, clearly not connected to the growth zone.

Plate ID	User	AB (software)	AB (user)
79	Intermediate	10.116	9.124
79	Experienced	4.765	9.619
79	Beginner	10.251	9.270

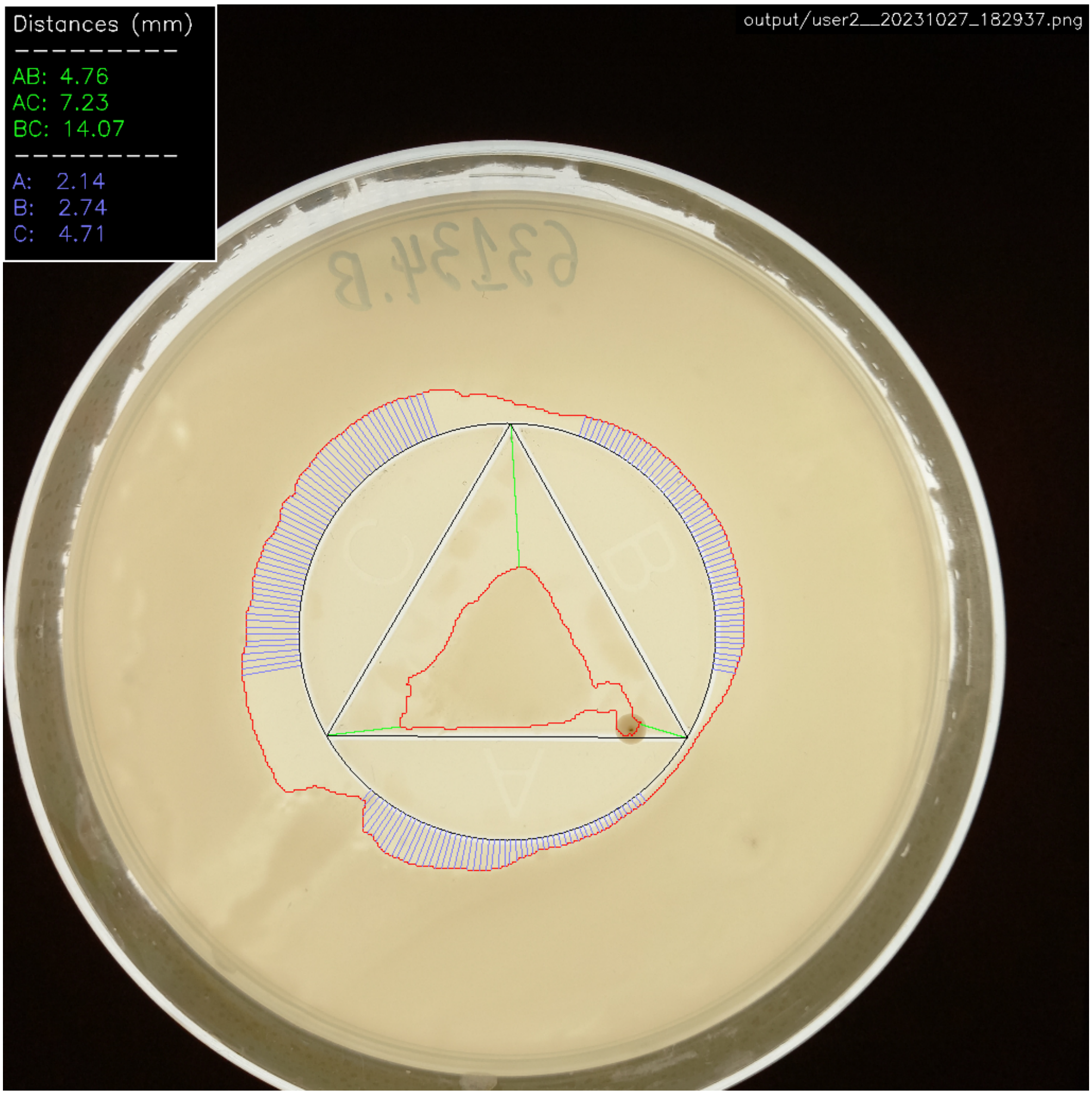

Outliers in plate 4

Clearly, the beginner user made a wrong assessment of distances AB and AC.

Plate ID	User	AB (software)	AB (user)	AC (software)	AC (user)
4	Intermediate	16.119	14.837	16.117	14.751
4	Experienced	17.036	14.982	15.727	14.642
4	Beginner	15.908	11.066	15.506	10.831

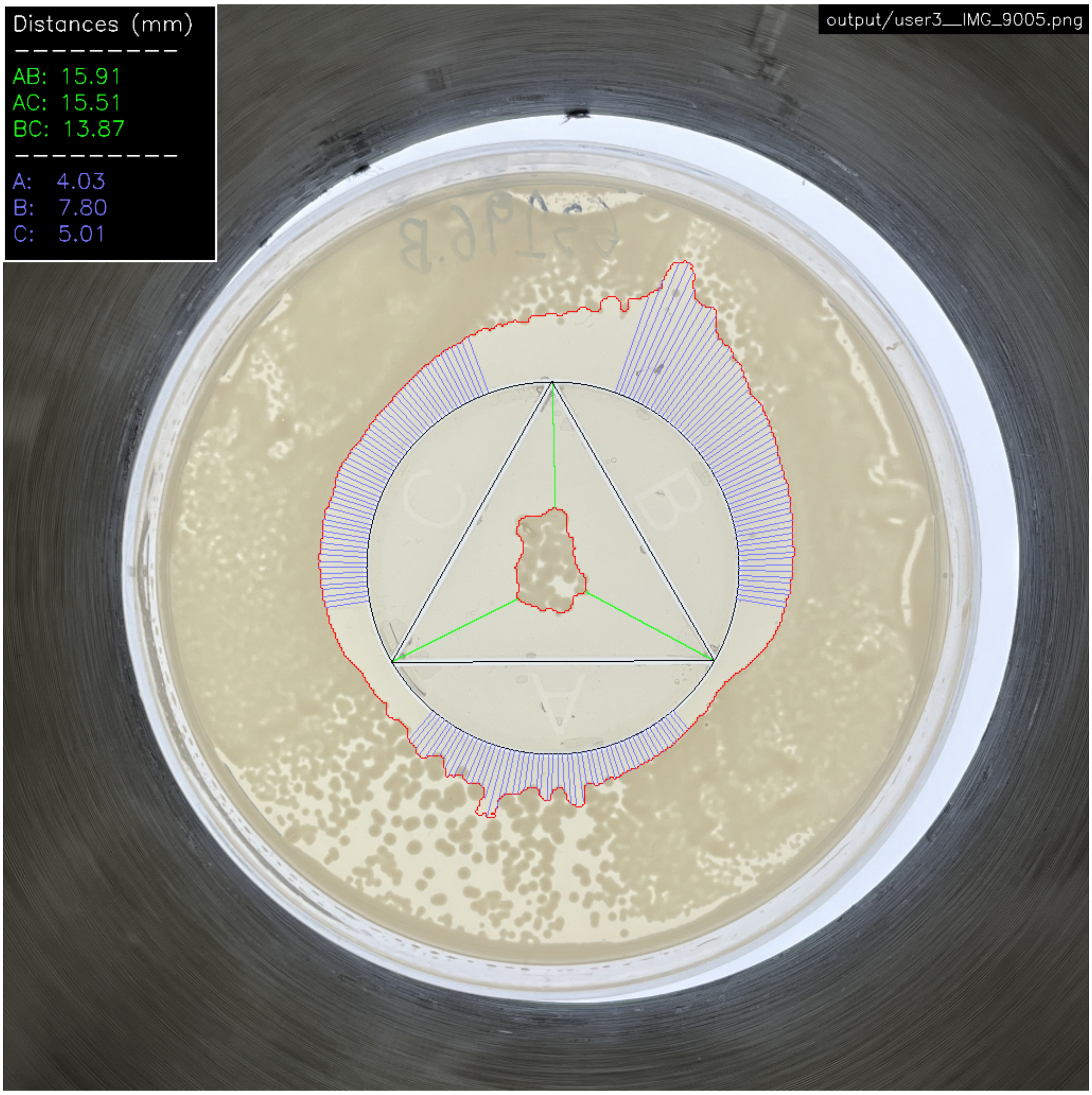

Supplement: S3 Appendix — All four plates where user and software measurements differed by more than 3.5 mm are visualized in one document, along with a possible explanation for the outliers. (PDF7) [file pdig.0000669.s003.pdf]
