# Supplementary figures and images for: CombiANT reader: Deep learning-based automatic image processing tool to robustly quantify antibiotic interactions
Source: PLOS Digit Health. 2025 Jul 8;4(7):e0000669. doi: 10.1371/journal.pdig.0000669 (PMC12237020; doi:10.1371/journal.pdig.0000669)

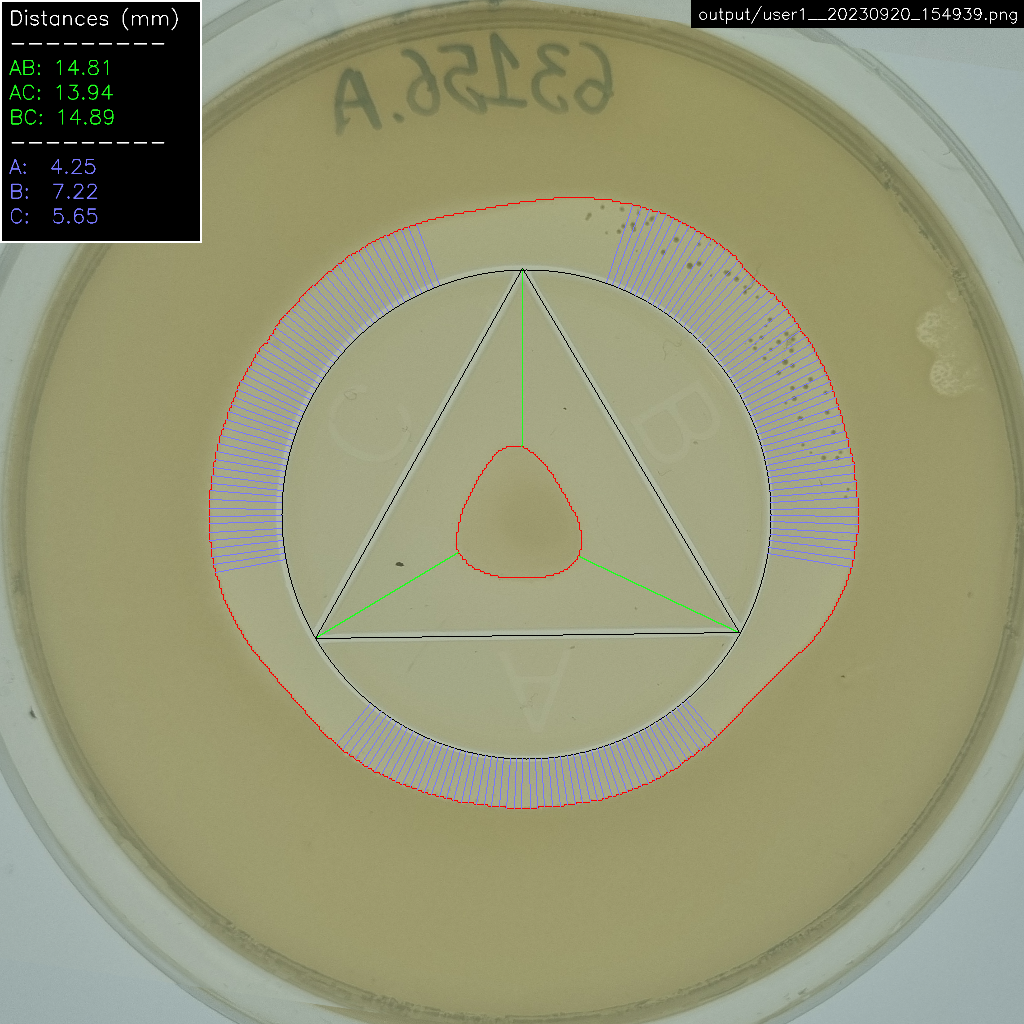

Supplement: S1 Fig — (PNG) [file pdig.0000669.s004.png]

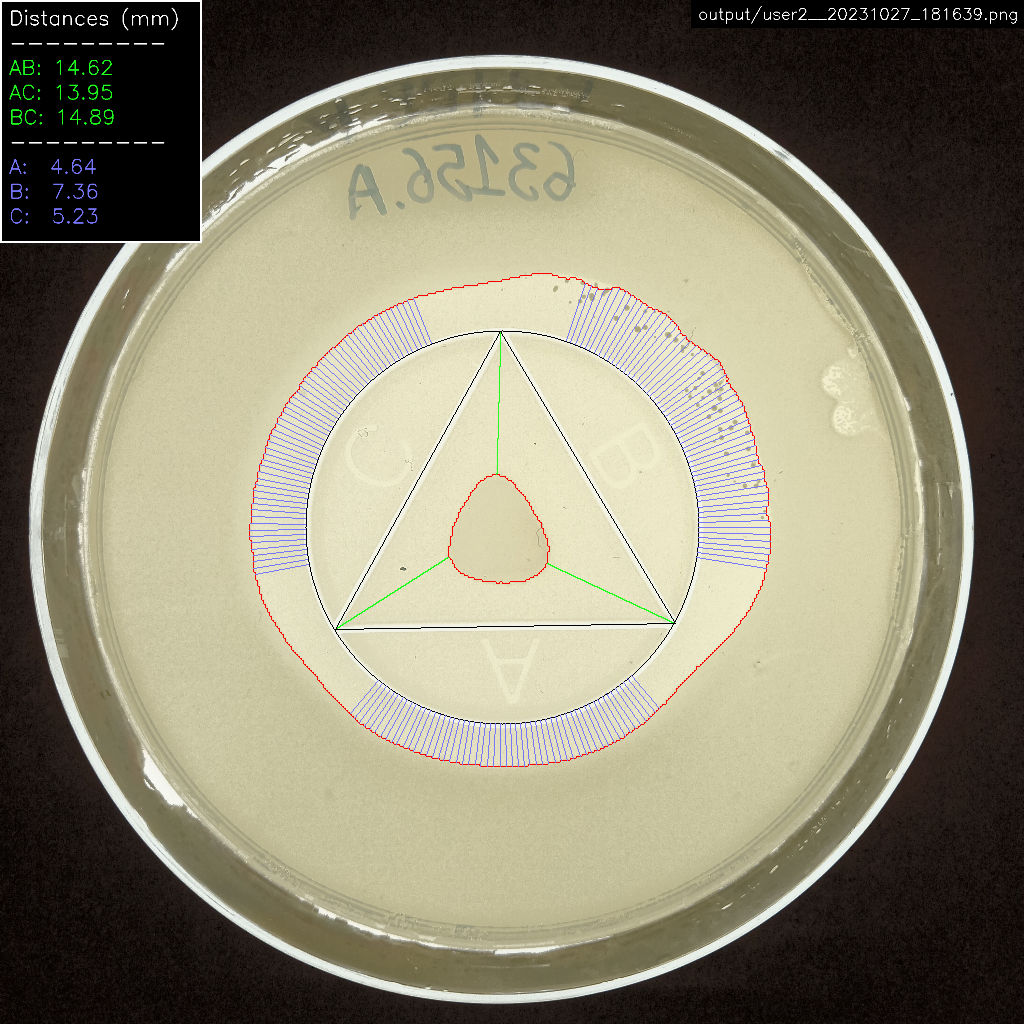

Supplement: S2 Fig — (PNG) [file pdig.0000669.s005.png]

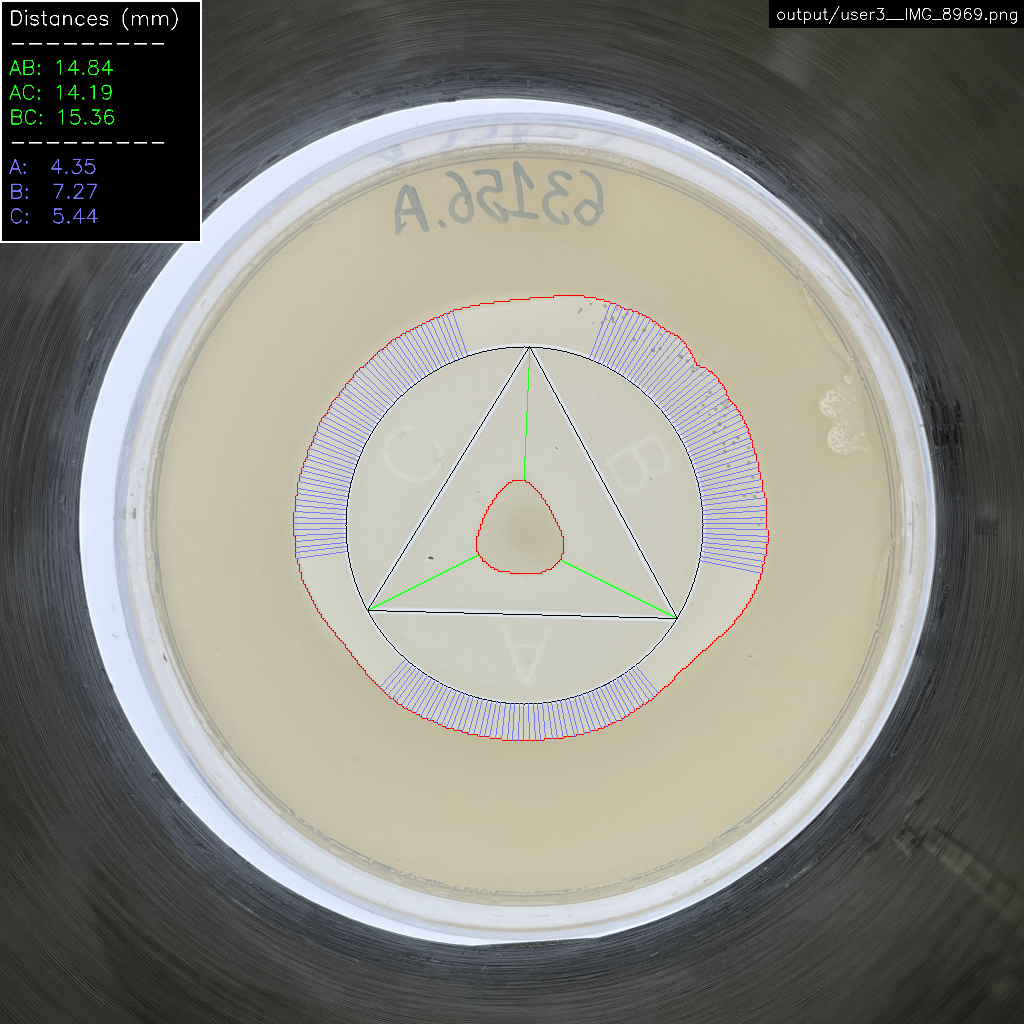

Supplement: S3 Fig — (PNG) [file pdig.0000669.s006.png]

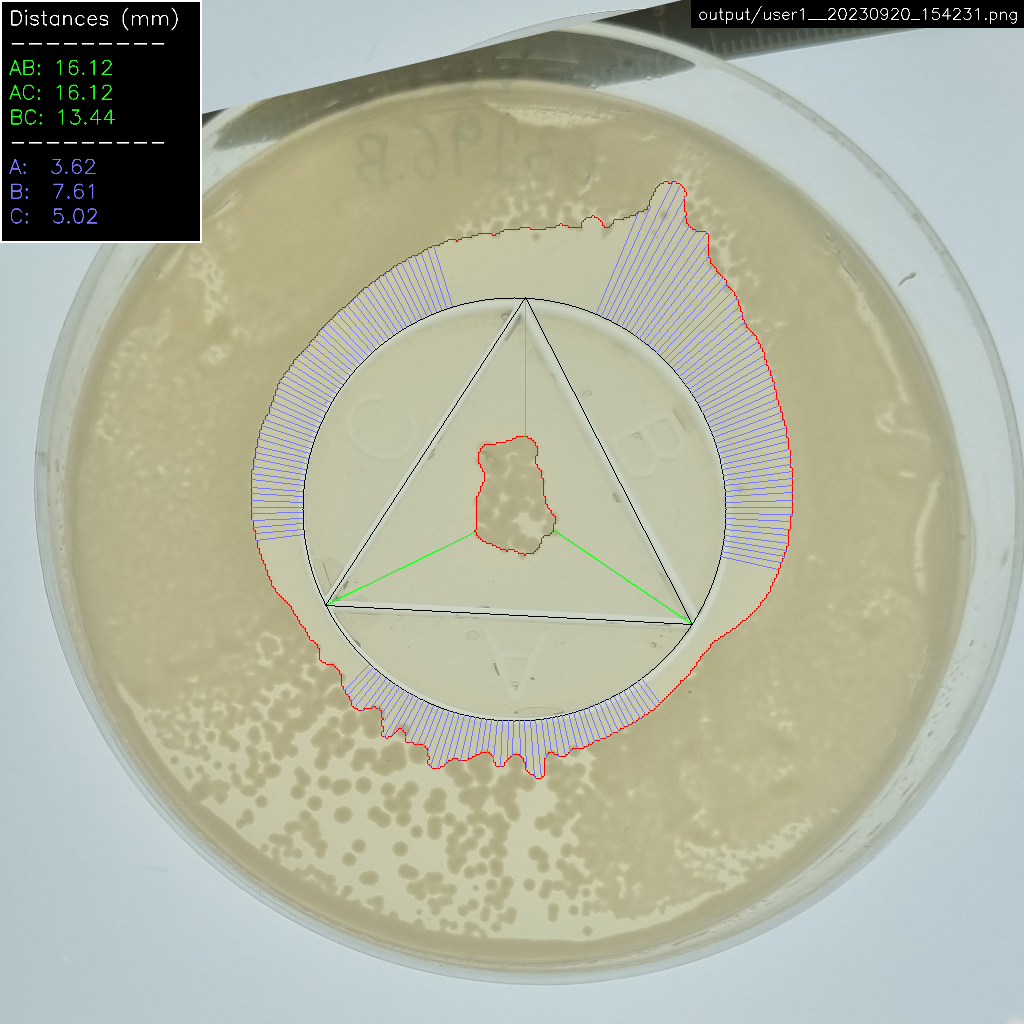

Supplement: S4 Fig — (PNG) [file pdig.0000669.s007.png]

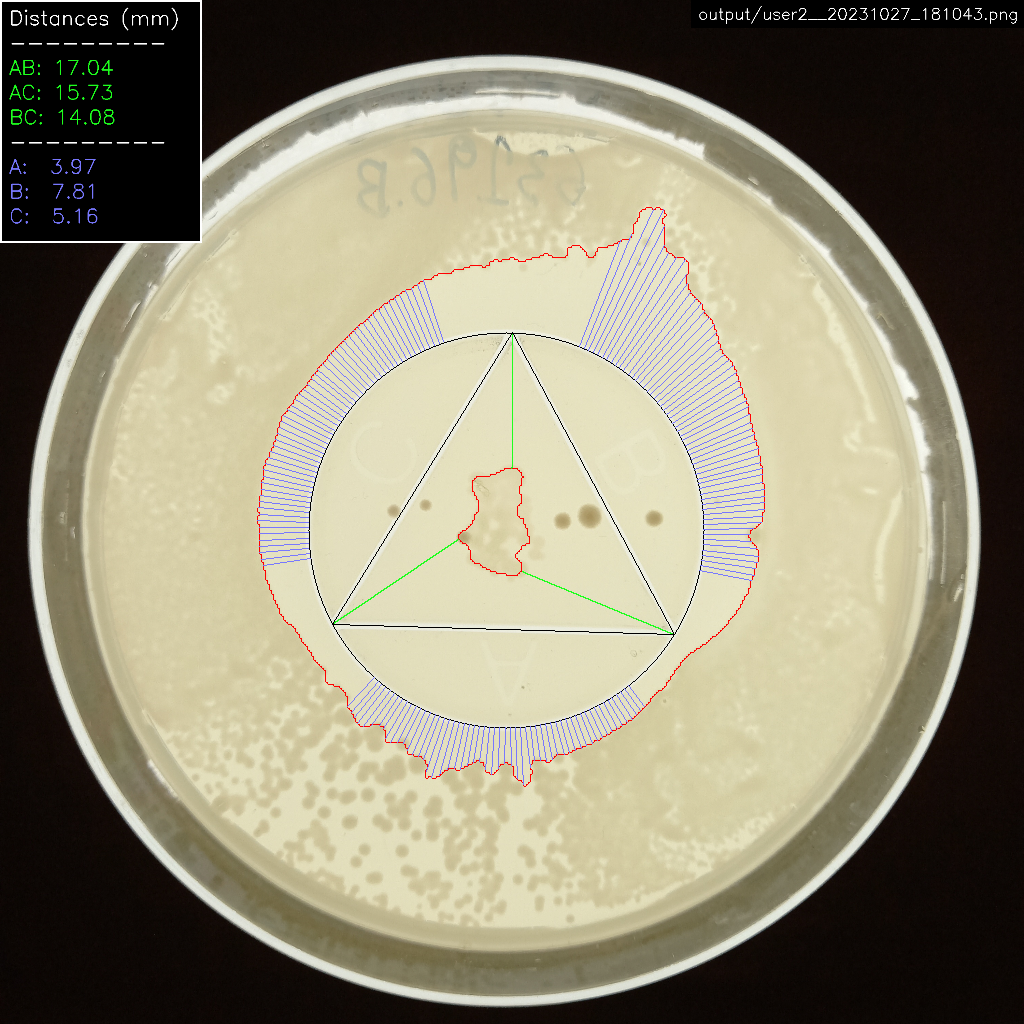

Supplement: S5 Fig — (PNG) [file pdig.0000669.s008.png]

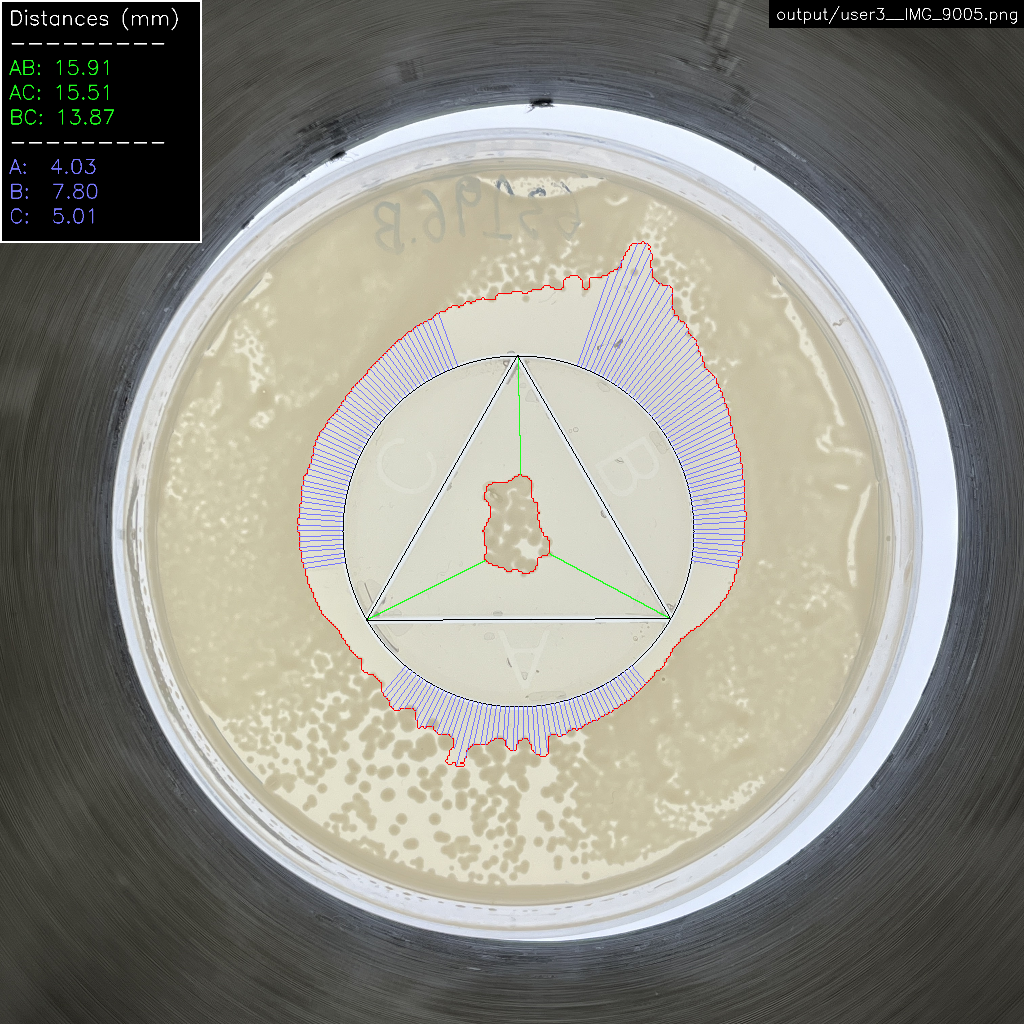

Supplement: S6 Fig — (PNG) [file pdig.0000669.s009.png]

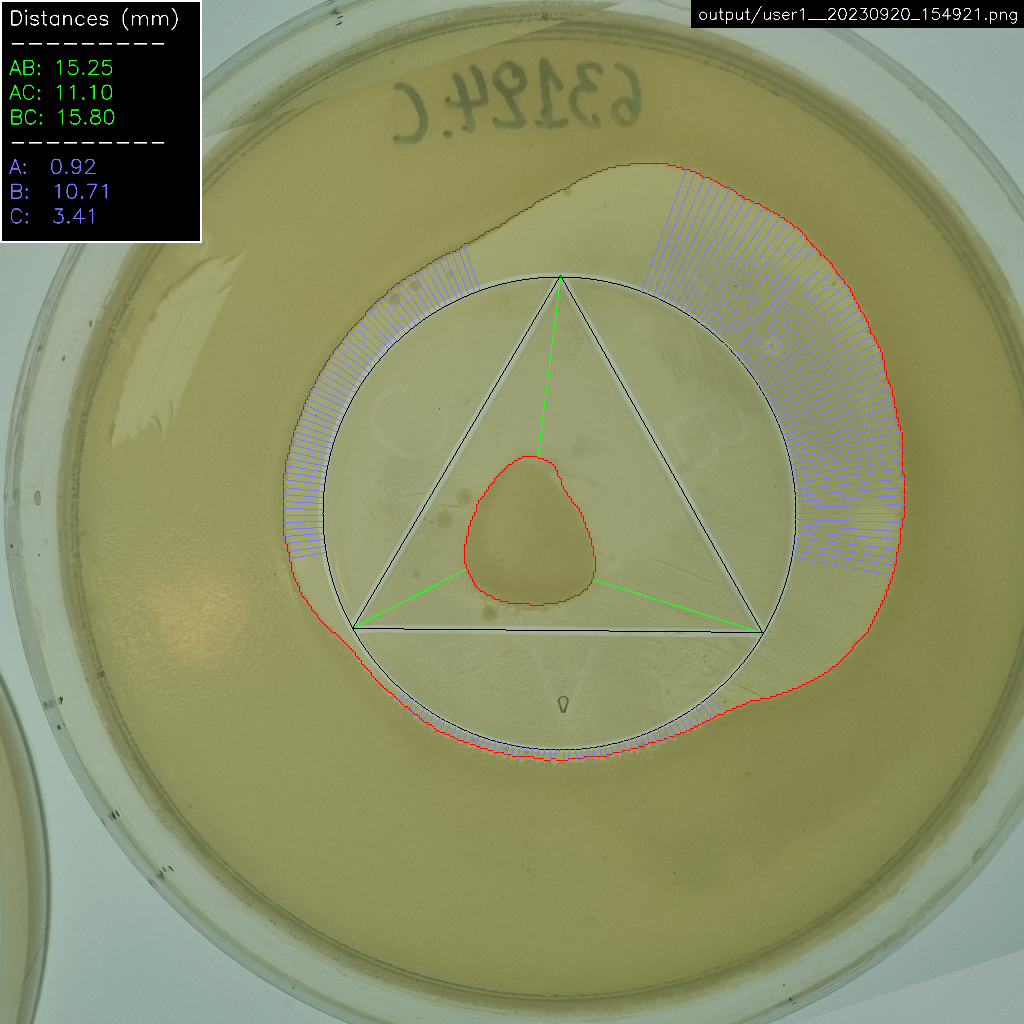

Supplement: S7 Fig — (PNG) [file pdig.0000669.s010.png]

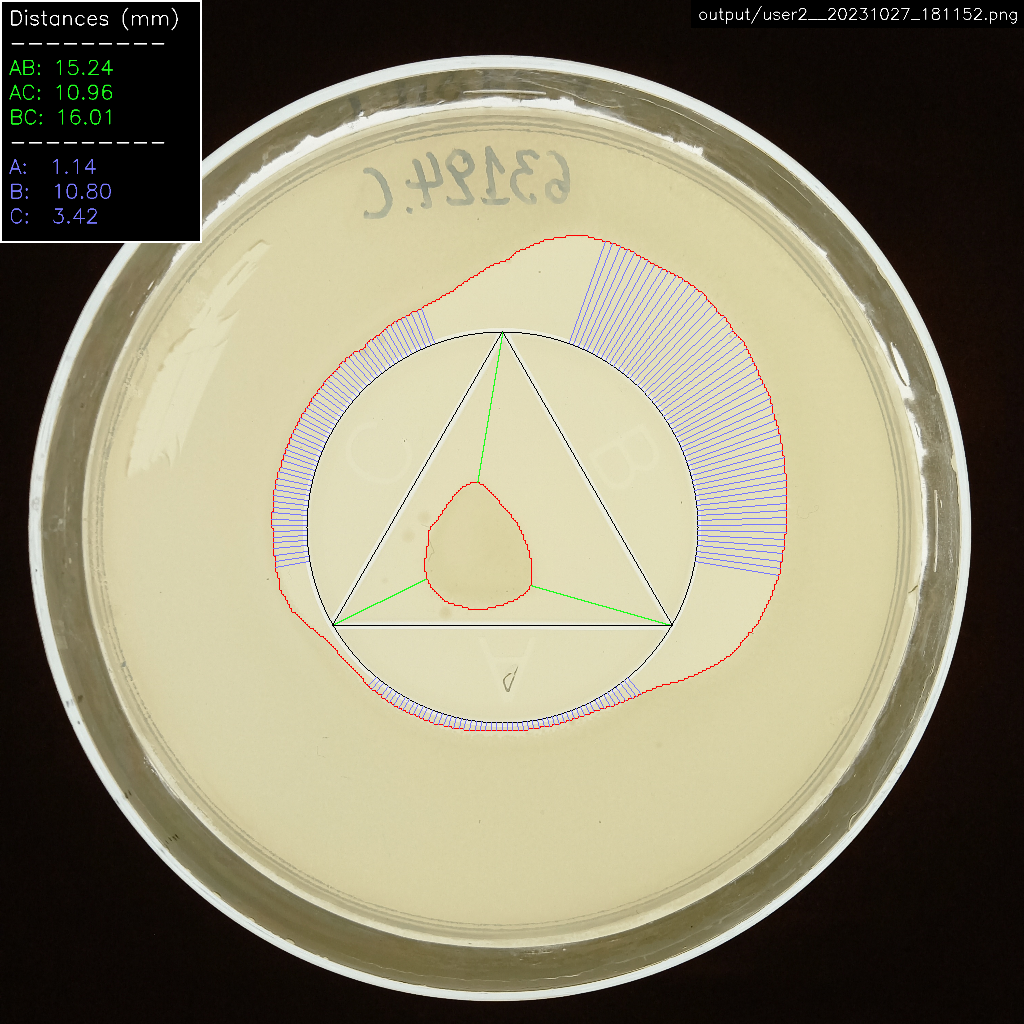

Supplement: S8 Fig — (PNG) [file pdig.0000669.s011.png]

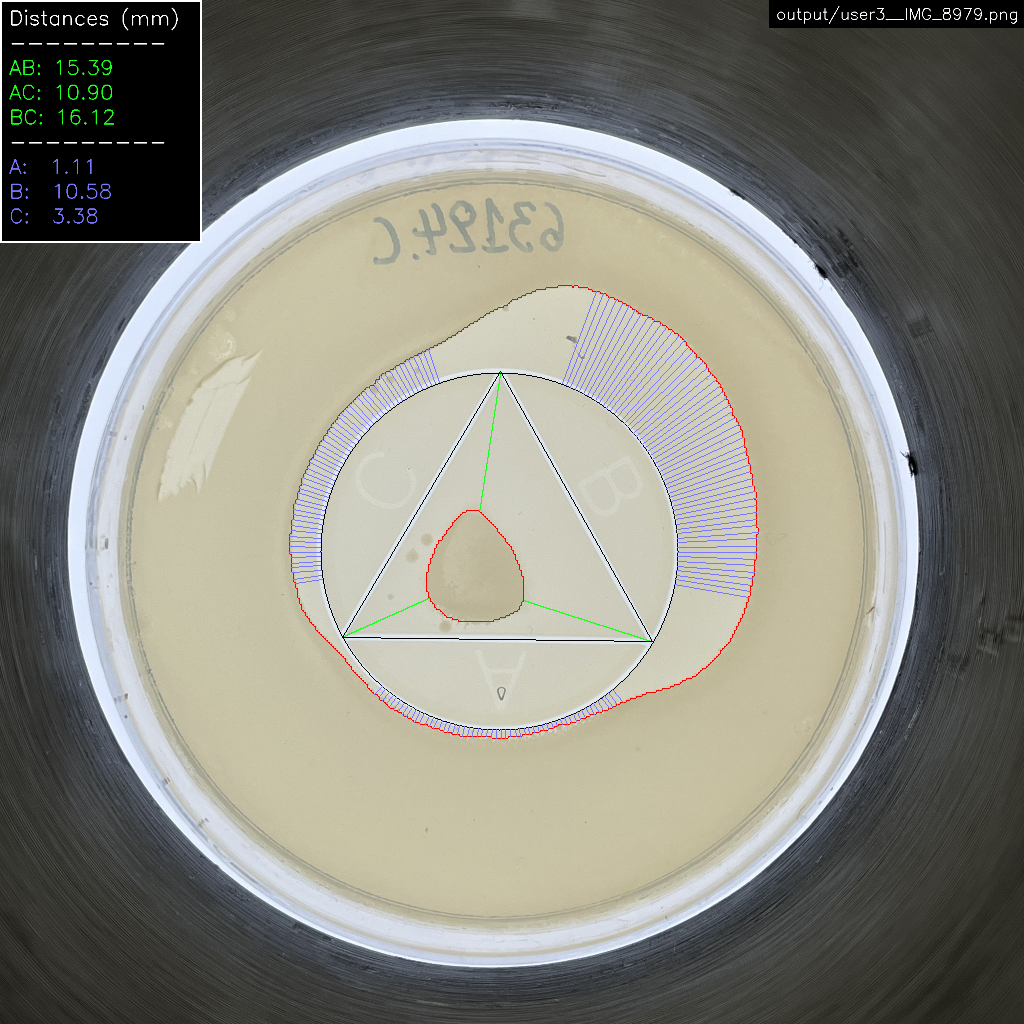

Supplement: S9 Fig — (PNG) [file pdig.0000669.s012.png]

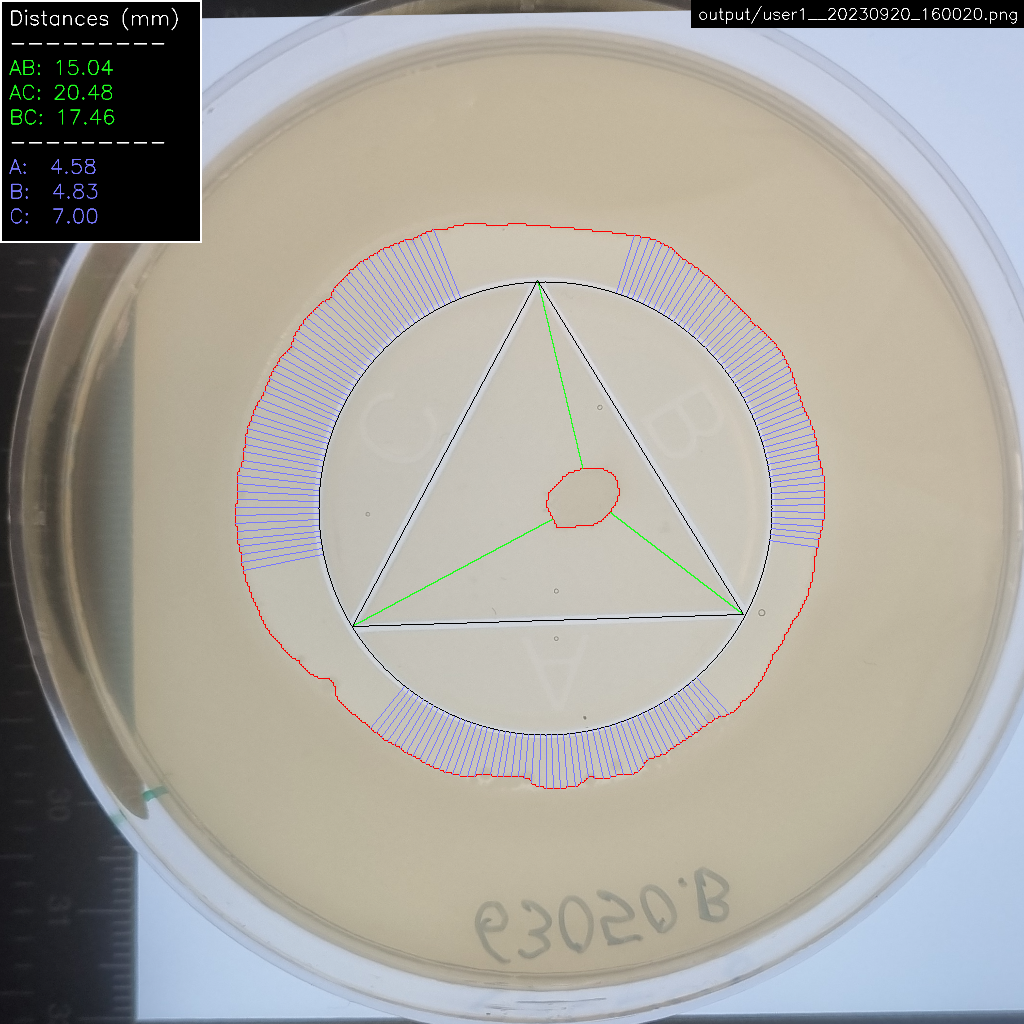

Supplement: S10 Fig — (PNG) [file pdig.0000669.s013.png]

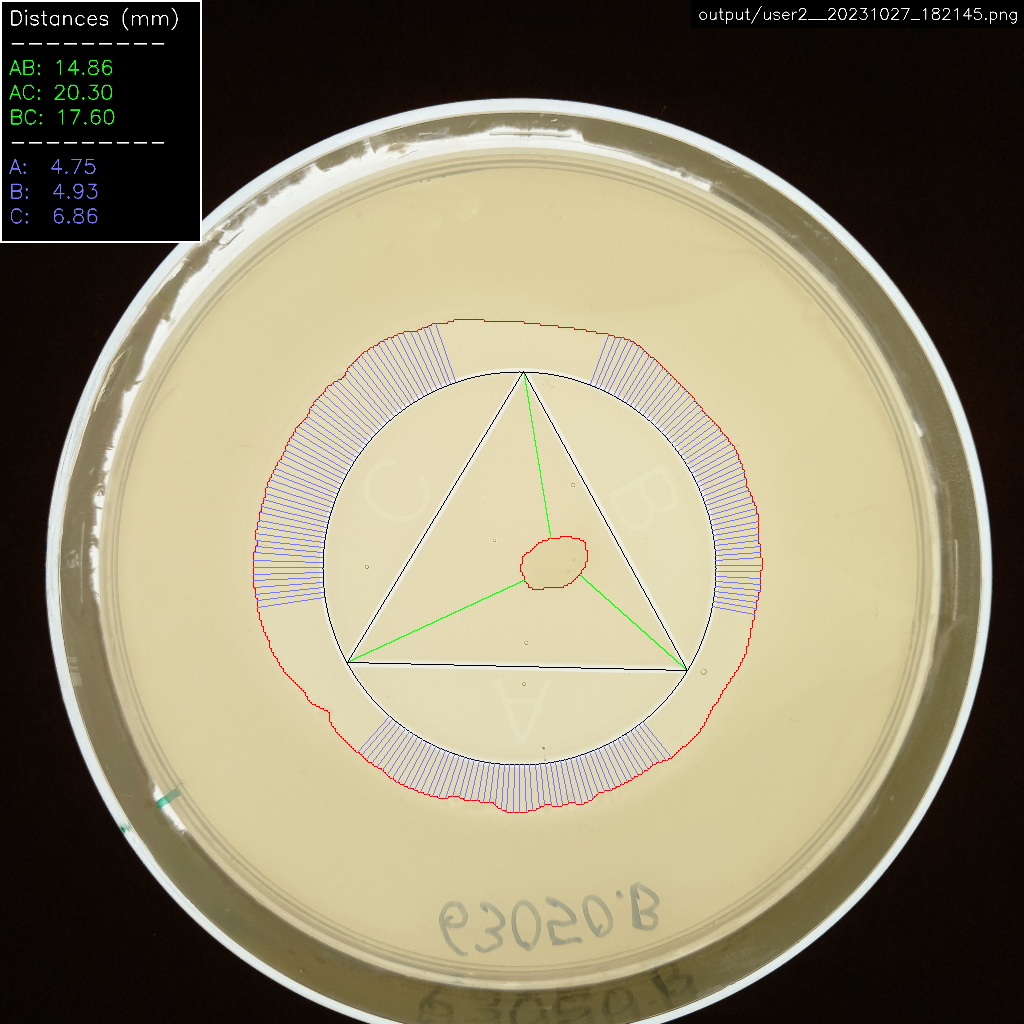

Supplement: S11 Fig — (PNG) [file pdig.0000669.s014.png]

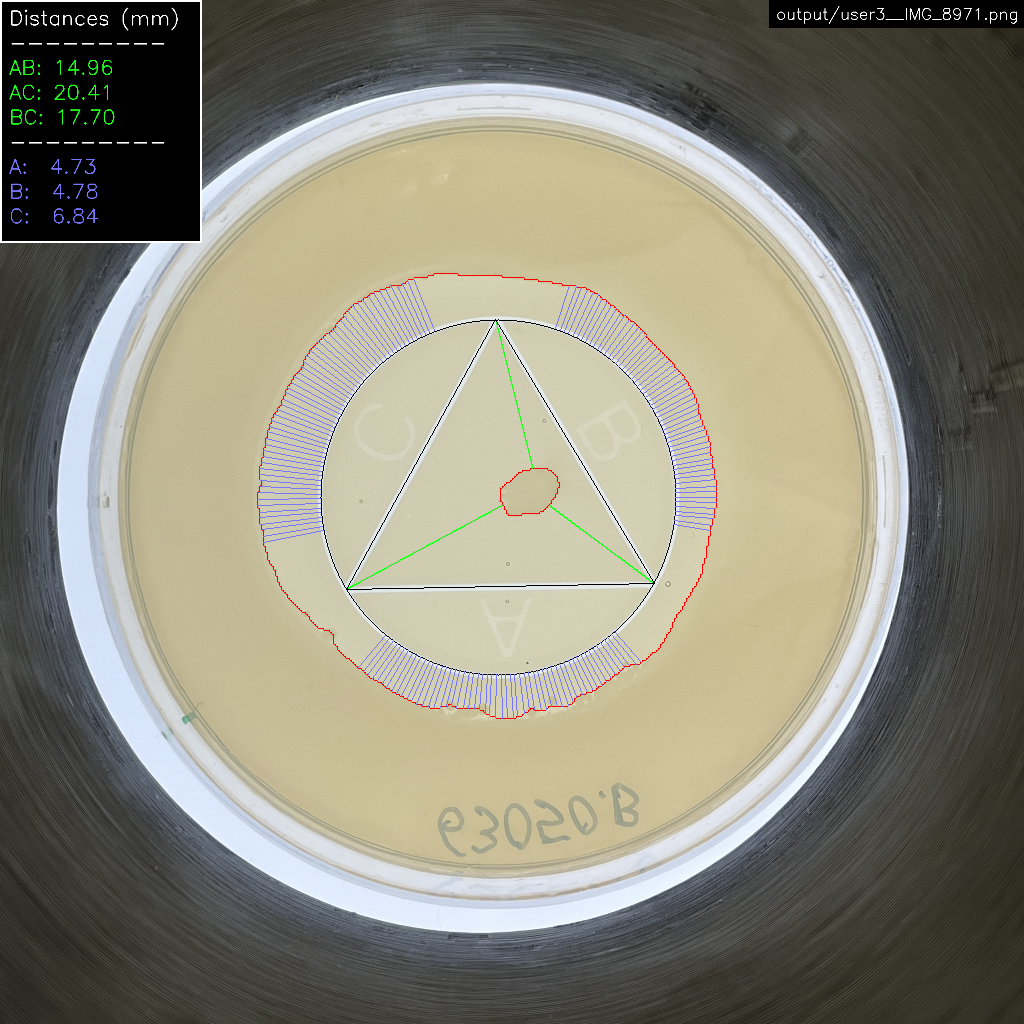

Supplement: S12 Fig — (PNG) [file pdig.0000669.s015.png]

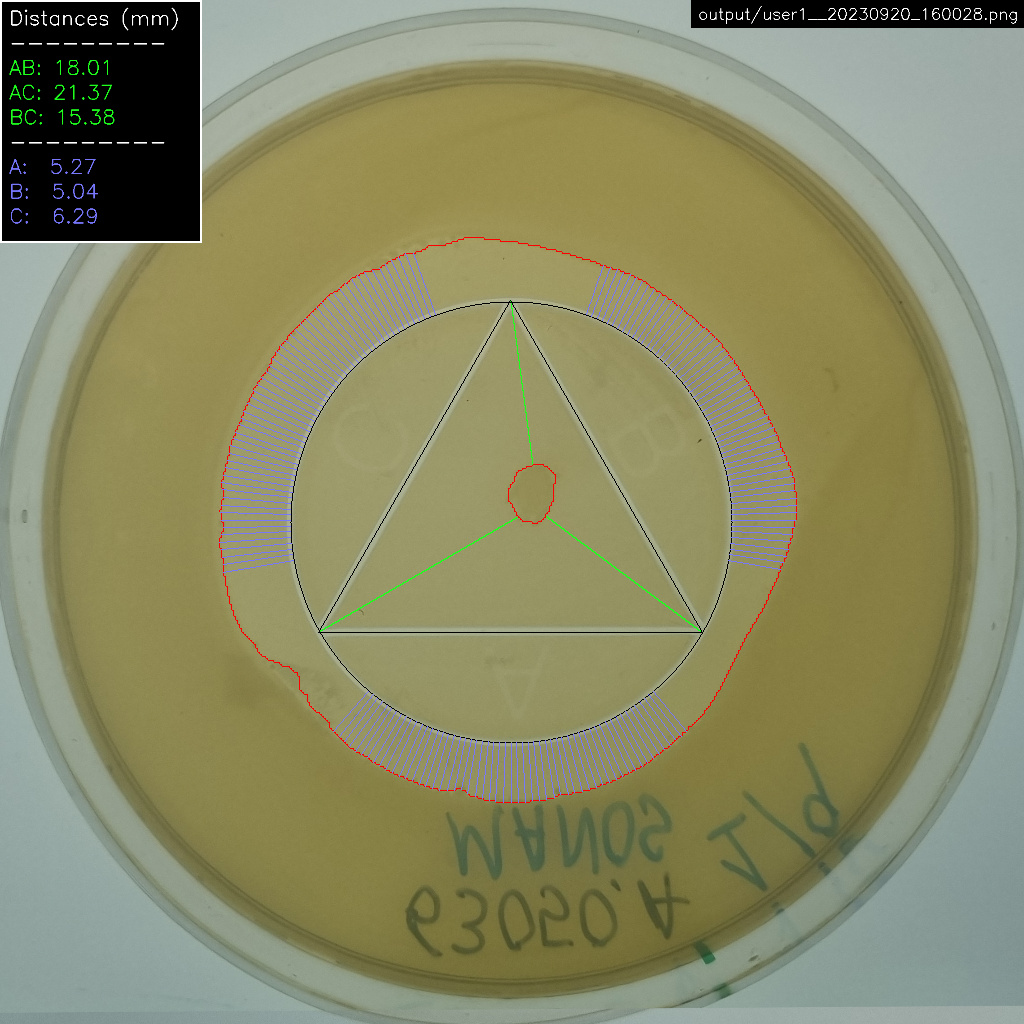

Supplement: S13 Fig — (PNG) [file pdig.0000669.s016.png]

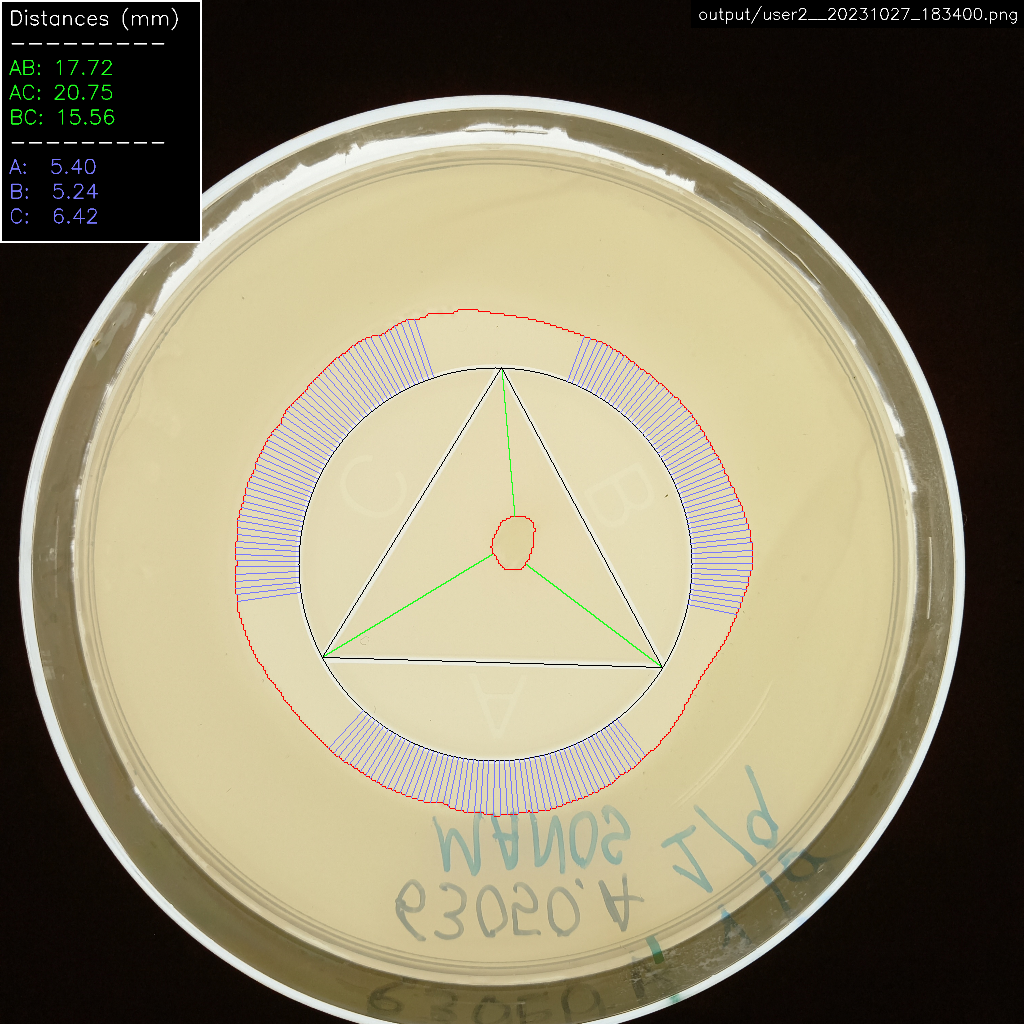

Supplement: S14 Fig — (PNG) [file pdig.0000669.s017.png]

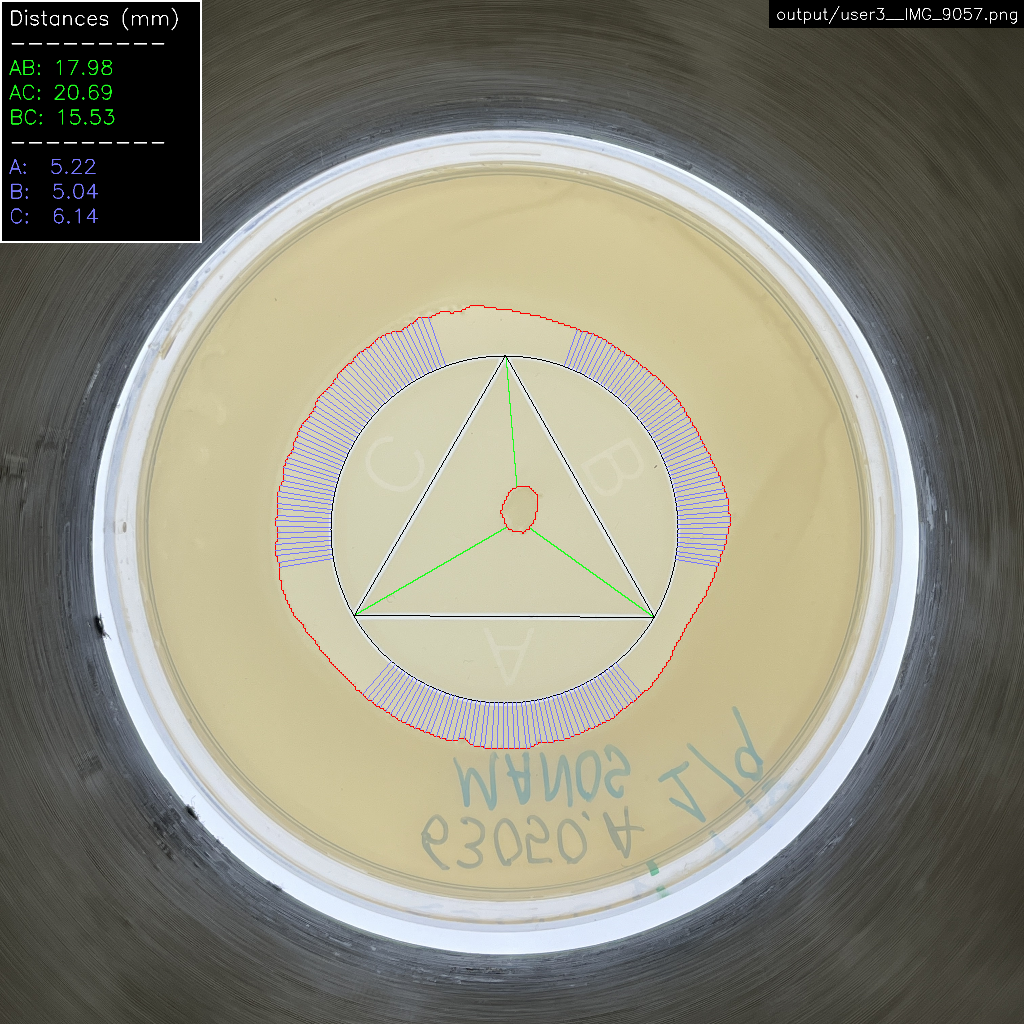

Supplement: S15 Fig — (PNG) [file pdig.0000669.s018.png]

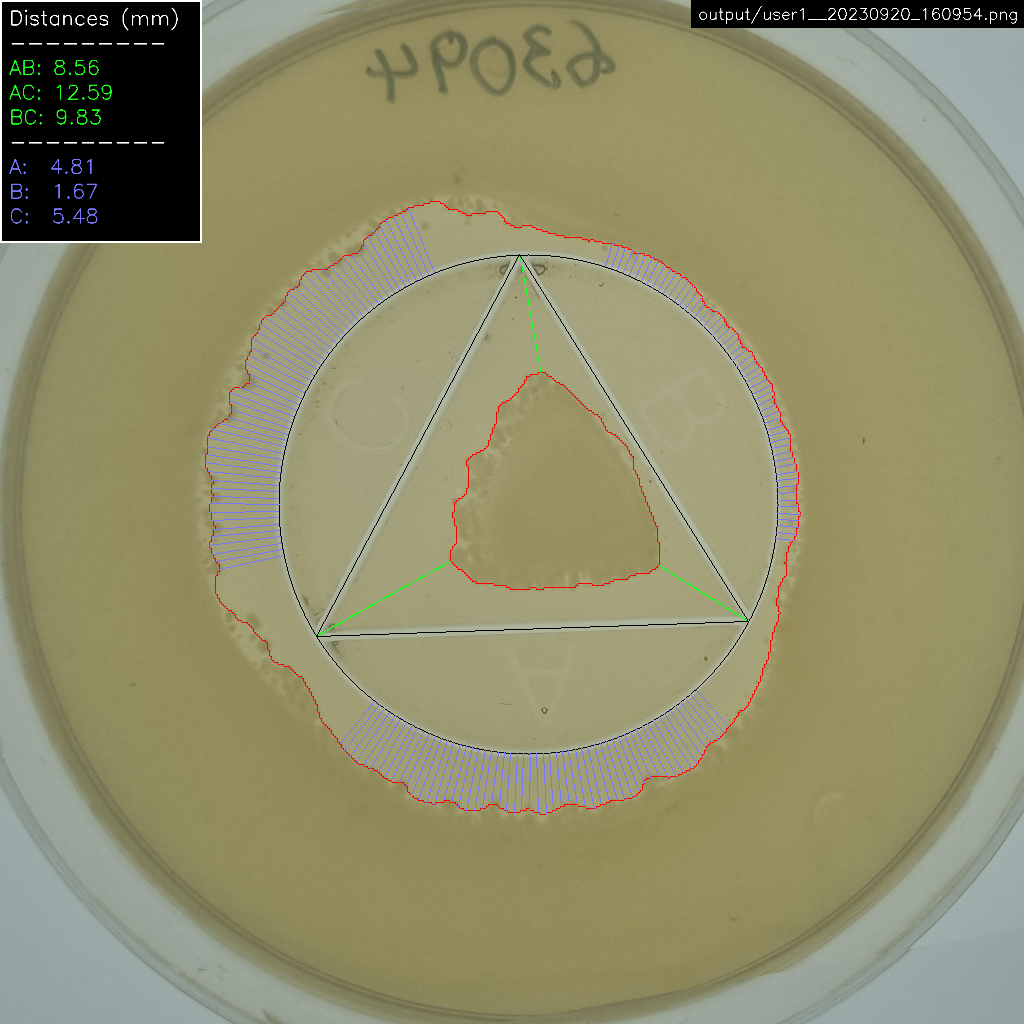

Supplement: S16 Fig — (PNG) [file pdig.0000669.s019.png]

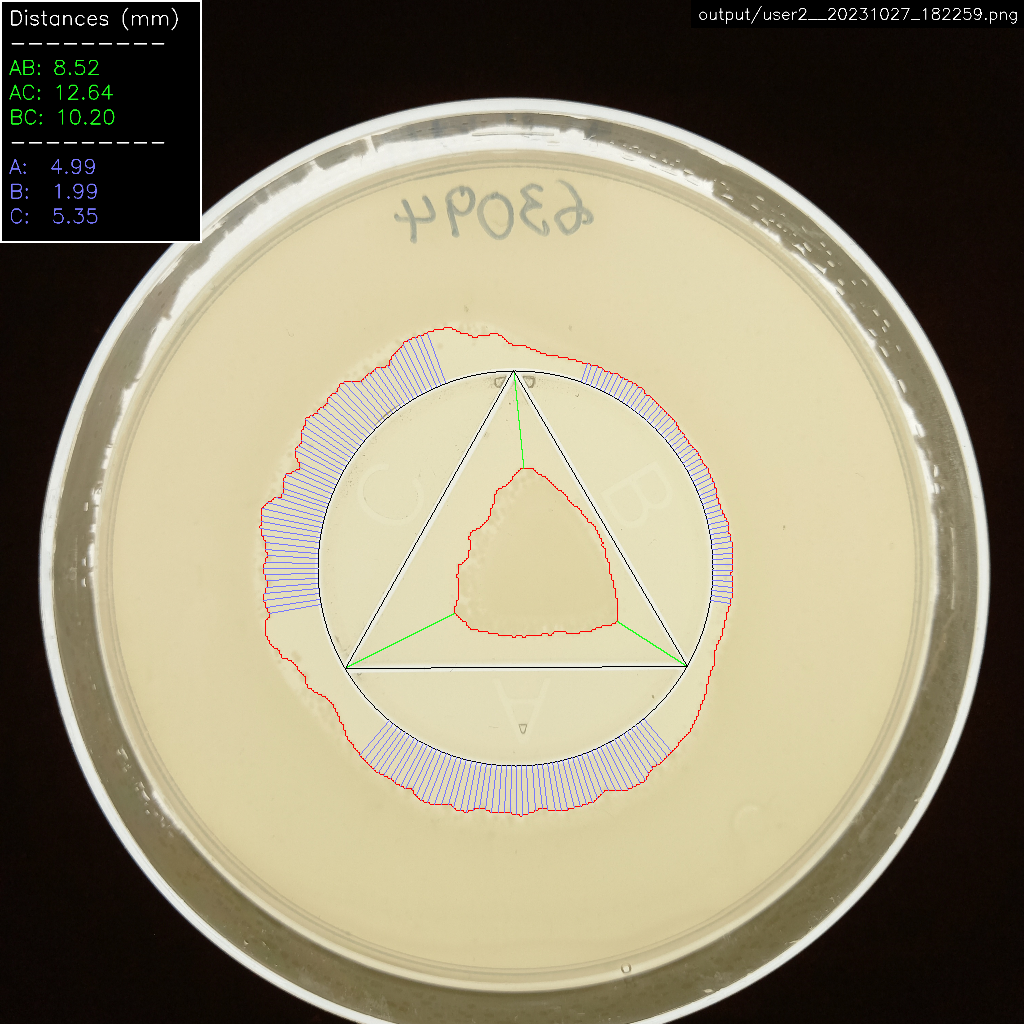

Supplement: S17 Fig — (PNG) [file pdig.0000669.s020.png]

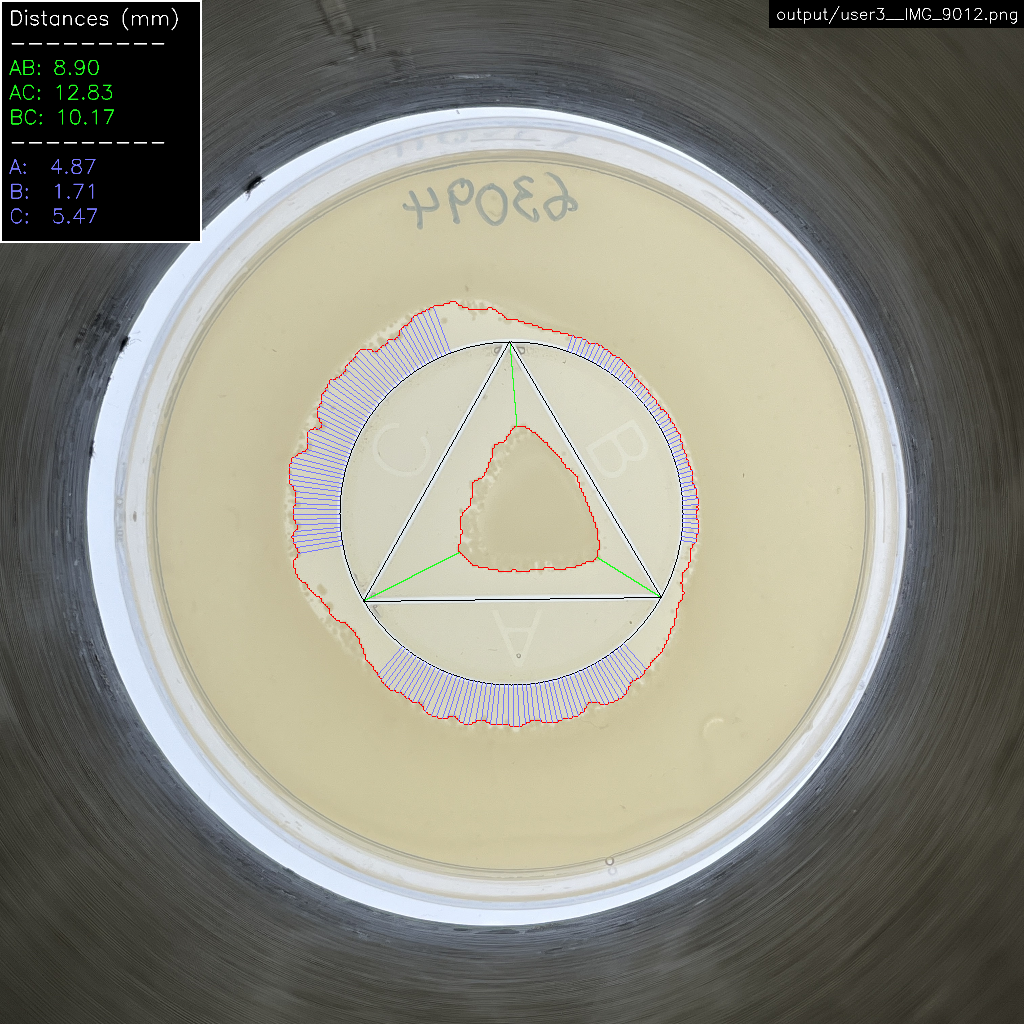

Supplement: S18 Fig — (PNG) [file pdig.0000669.s021.png]

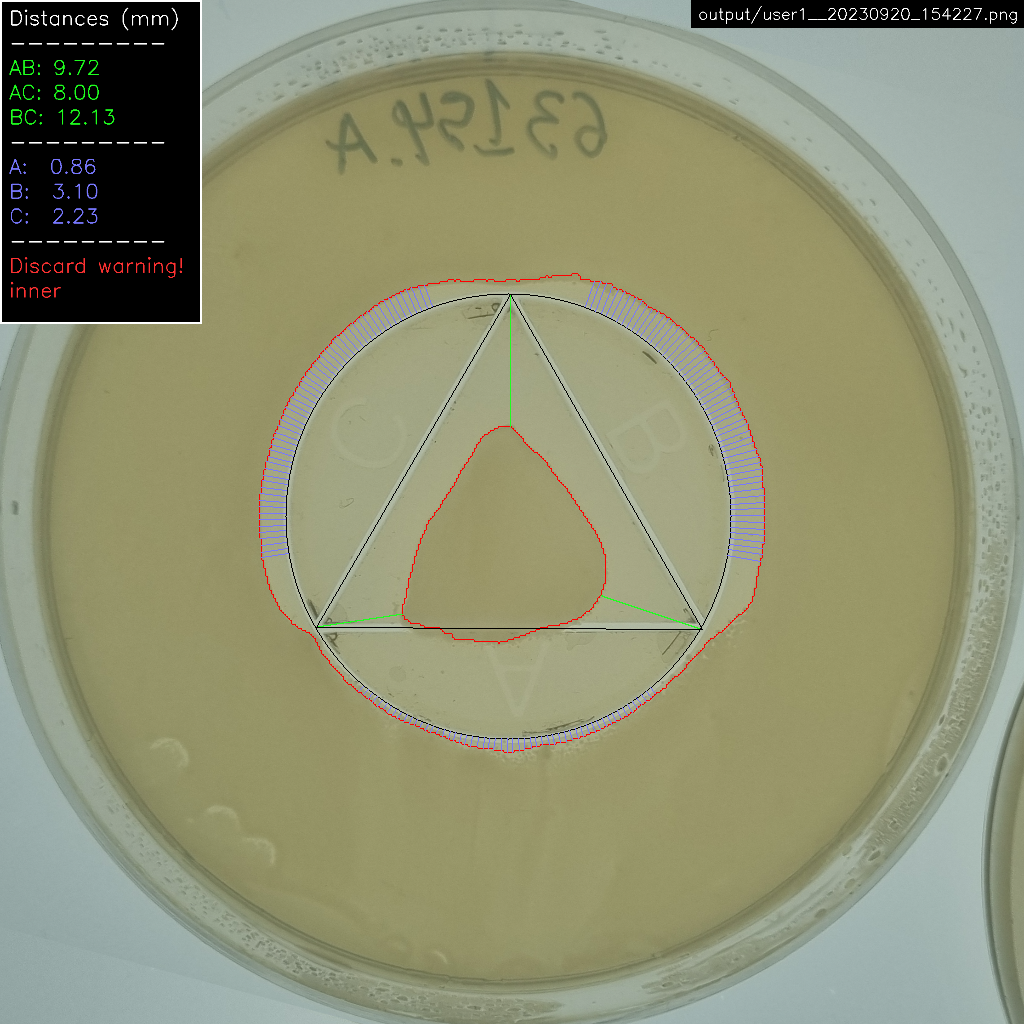

Supplement: S19 Fig — Discarded plate due to inner growth zone intersecting with the triangle-mark. (PNG) [file pdig.0000669.s022.png]

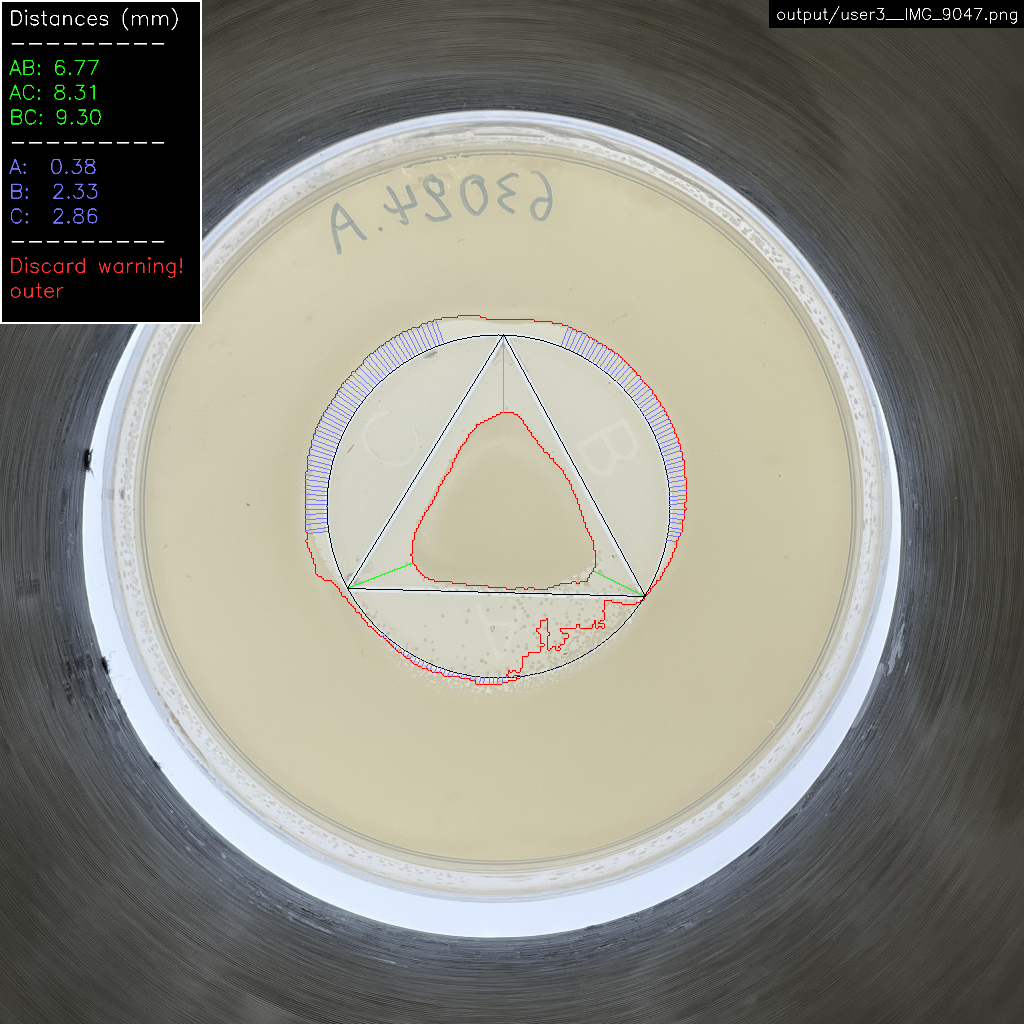

Supplement: S20 Fig — Discarded plate due to the outer growth zone growing past the circle mark. (PNG) [file pdig.0000669.s023.png]
