# Supplementary material for: CombiANT reader: Deep learning-based automatic image processing tool to robustly quantify antibiotic interactions
Source: PLOS Digit Health. 2025 Jul 8;4(7):e0000669. doi: 10.1371/journal.pdig.0000669 (PMC12237020; doi:10.1371/journal.pdig.0000669)
Supplement: Attachment — Submitted filename: Response CombiANT Reader.pdf [file pdig.0000669.s024.pdf]

Response to reviewers

Dear reviewers and editors,

Thank you for your insightful comments and valuable feedback on our study.
Reviewer comments are in dark blue with our response right below in black.

Main changes:

- The original plots used GraphPad Prism, and displayed the median values with error bars showing a 95% confidence interval (hence the asymmetry). This has been changed to standard deviation and mean plotted using Matplotlib in Python.
- Plots for the inner distances were added in the Results section.
- Table 1 was added with quantitative metrics in the Results section.
- Carolina Wählby was added as co-author for funding acquisition.
- One “Outliers” supporting document showing plates with distances where the user and the software assessment differed by more than 3.5mm
- One “Discarded plates” supporting document showing all plates flagged for discarding.
- One “CombiANT” supporting document giving a detailed explanation of CombiANT and the explanation in the introduction has been simplified.

Reviewer #1

- The title of the article, "CombiANT Reader - Deep learning-based automatic image processing and measurement of distances to robustly quantify antibiotic interactions," effectively conveys the critical focus and novelty of the study, which lies in utilizing deep learning for automated image analysis in the context of antibiotic interactions. However, I would recommend another version of the title for more precision and clarity, such as : “CombiANT Reader: A Deep Learning-Based Tool for Automatic Image Processing and Quantification of Antibiotic Interactions Using Distance Measurement”

The title was changed to *“CombiANT Reader: Deep learning-based automatic image processing tool to robustly quantify antibiotic interactions”*

The word “tool” was added, putting “robustly quantify antibiotic interactions” in the end because that is the main goal of the software. The distance measurements are only one part for the pipeline, which also contains template matching and segmentation.

The abstract:

- The abstract is well formed with an apparent problem statement, the relevance of the research question under study, with novel methods, and showed good validation and consistency; however, I have some recommendations that may enhance it; these are:

- Quantify Performance: Include specific metrics to highlight the software's accuracy and reliability compared to human scoring.
  The quantitative performance metric mean absolute error between user and software measurements was calculated and added in the abstract.

- Expand Practical Implications: Briefly discuss how the method could impact clinical workflows or large-scale research on antibiotic resistance.
  A paragraph discussing clinical workflows and large-scale research of antibiotic resistance was added in the abstract.

- Clarify Technical Details: Concisely explain "150 distances" to enhance comprehension.
  The sentence was changed to “distances between key points” as the exact number of (outer) distances can be adjusted and is not essential here.

- Refine Language: Avoid repetitive phrasing and improve the flow for readability.
  We revised the abstract sentence by sentence, and each contains important information about the study.

The introduction:

- Although it has strong points as follow:

- The introduction clearly outlines the global threat of antibiotic resistance and highlights the importance of evaluating antibiotic interactions, particularly for combination therapies. This sets a strong foundation for the study.

- The detailed explanation of the CombiANT assay, including its setup, diffusion dynamics, and manual scoring process, provides valuable context for understanding the study's focus.

- The introduction identifies a significant gap in the lack of automated scoring methods for the CombiANT assay, effectively justifying the development of the proposed deep learning-based solution.

- By referencing related methods such as the Kirby–Bauer disk diffusion test and colony-forming unit (CFU) counting, the introduction situates the study within the broader context of antibiotic testing methodologies.

- I think some weaknesses could be strengthened for better readability and clear ideas, such as:

- Complexity and Redundancy: The introduction is dense with technical details, which may overwhelm readers unfamiliar with the CombiANT assay. Specific descriptions,

such as the manual scoring process and triangle vertex annotations, are overly detailed for an introduction and could be summarized.
The technical details were moved to a supporting document, and a brief simplified explanation of CombiANT was added instead (lines 14-25).

- Limited Focus on Study Objectives: While the introduction provides ample background, the study’s objectives and potential impact are not emphasized. A stronger focus on how this pipeline advances antibiotic resistance research would strengthen the narrative.
  A paragraph highlighting the study’s overall objective and potential impact was added, including how it could advance antibiotic resistance research on line 75: *“The overall objective of the study is to demonstrate…”*

- Lack of Transition to Deep Learning: The introduction transitions abruptly from explaining the manual CombiANT test to discussing the deep learning-based pipeline. A smoother connection highlighting why deep learning is a suitable solution would improve coherence.
  A paragraph has been added discussing recent advances in image processing using deep learning and why using it was a suitable solution starting from line 26.

- Insufficient Highlighting of Novelty: Although the study’s novelty is implied, it is not explicitly stated. Emphasizing what distinguishes this work from existing methods (e.g., precision, scalability, or adaptability to non-circular inhibition zones) would make the contribution more straightforward.
  A section has been added distinguishing the work from existing methods. The novelty is explicitly stated *“At present, no computer vision application for automatically scoring the CombiANT assay is available”* (line 37), and *“To address this lack of automated analysis of antibiotic interactions, an image processing pipeline was developed in this study for this specific application.”* (line 50)

My Recommendations for the results section are:

- Include Quantitative Validation: Add specific quantitative metrics such as correlation coefficients, mean absolute error, or Bland-Altman analysis to strengthen the argument for the software's accuracy.
  Table 1 has been added in the results sections, presenting the mean absolute error between the user and software gradings, with a breakdown on user and distance, along with the mean MAE for each distance (averaged across all users) and mean MAE for each user (averaged across all distances).

- Clarify Outliers and Plate Discarding: Expand on the reasons for outliers and provide examples of the criteria used for plate discarding to ensure transparency.
  A supporting document has been added showing outliers and the possible explanations. The plate discarding has been clarified, and stated that these plates

were still part of our evaluation since containing useful distances. A supporting document has been added showing all plates flagged for discarding.

- Discuss Intermediate User Performance: Highlight the intermediate user's performance to understand better how the software performs across varying skill levels.
  We are unsure how to highlight the intermediate user's performance, main takeaway of the results is that the software performs comparably to human users across different skill levels.

- Expand on Software Advantages: While reliability and consistency are emphasized, the results should also discuss the software's potential time savings and scalability benefits compared to manual scoring.
  A sentence has been added emphasizing potential time savings and scalability on line 87.

My suggestions and comments for the discussion section:

Although the discussion section has lots of information and is well formed in a clear and informative way, these are my comments that may enhance it and make it more precise and more profound:

- It lacks Quantitative Performance Metrics: The discussion refers to the software's "consistency with human evaluation" but lacks concrete metrics to substantiate this claim. Including statistical results (e.g., accuracy, precision, recall) would make the argument more robust.
  The mean absolute error between the user and software has been added in Table 1. We are unsure how to present accuracy, precision and recall as these typically are classification metrics.

- It has Limited Exploration of Scalability: While the discussion mentions cloud-based analysis, it does not elaborate on how the software can be scaled for high-throughput settings, which could enhance its applicability for larger clinical or research facilities.
  A sentence has been added in the discussion highlighting scalability in line 146.

- It includes a Superficial Description of Future Optimizations: The potential use of transformer architectures and other advanced models is not explored in depth. A brief explanation of how these advancements could address specific challenges (e.g., rotation, perspective effects) would add value.
  We have elaborated on using Vision Transformer for segmentation in line 174. Main future improvement would be to switch out the template matching part to using keypoint estimation, a possible approach outlined in from line 159.

- It has an Overgeneralization of Future Impact: The statement about inspiring future work is broad. Adding concrete examples or potential areas where the software could be extended (e.g., analyzing other types of agar-based assays or antimicrobial resistance studies) would make this vision more compelling.
  Extension to other possible agar-based assays have been added on line 183.

My Recommendations for the Materials and Methods section:

- Justify Parameter Choices: Include brief explanations for key parameter selections and how they were optimized during development to add credibility to the methodology.
  The choice of parameters for the the deep learning model training were outlined in line 212, we used standard parameters and performed no hyperparameter optimization.

- Enhance Training Data Description: Provide more details on the diversity of the training dataset (e.g., types of bacteria, variations in assay design) to help readers assess the model's generalizability.
  There was a low diversity in the training set, photos taken straight above with high contrast, as written from line 218 we were able to get the model to work by introducing heavy augmentation. In a real setting, one would not treat this as a static model, rather continuously obtain real data, annotate and retrain the model. As written in line 143: *“If the segmentation fails, more data can easily be annotated, followed by retraining the network”*

- Automate Preprocessing: Highlight future plans to automate manual preprocessing steps using deep learning models or image normalization techniques, such as contrast adjustment or rotation.
  In a real setting with continuously obtaining training data annotating and retraining the network one would not require image normalization or contrast adjustment. One possible strategy to switching out the template matching has been outlined in the Discussion on line 159.

- Discuss Scalability: Expand on how the system could be scaled for high-throughput applications, including computational requirements for cloud deployment.
  There should be no scalability issues, as outlined in the Discussion on line 146, preparing the plates and the and experiments would be the time bottleneck.

- Analyze User Bias: Conduct a systematic analysis of user bias by evaluating the impact of user expertise and image quality on segmentation and distance measurements.
  We are not sure how to accomplish this. However, we can see from Table 1 and Figs 7-9 that the automated analysis systematically overestimates the inner distances compared to the users (especially for the BC triangle vertex pointing upwards). Our hypothesis is when the users are putting the triangle points, they are not putting them exactly at the corner of the triangle but rather on the inside edge of the triangle. As seen in Fig 9, this overestimation is lower for experienced users. The systematic error is about 1mm, about 10 pixels with the resolution 0.0935 +- 0.15 mm/pixel (wich was calculated for all assays). As seen in the images below, the distances estimated by the software exactly align with the center of the circle. We would like to limit the study to the current user comparison.

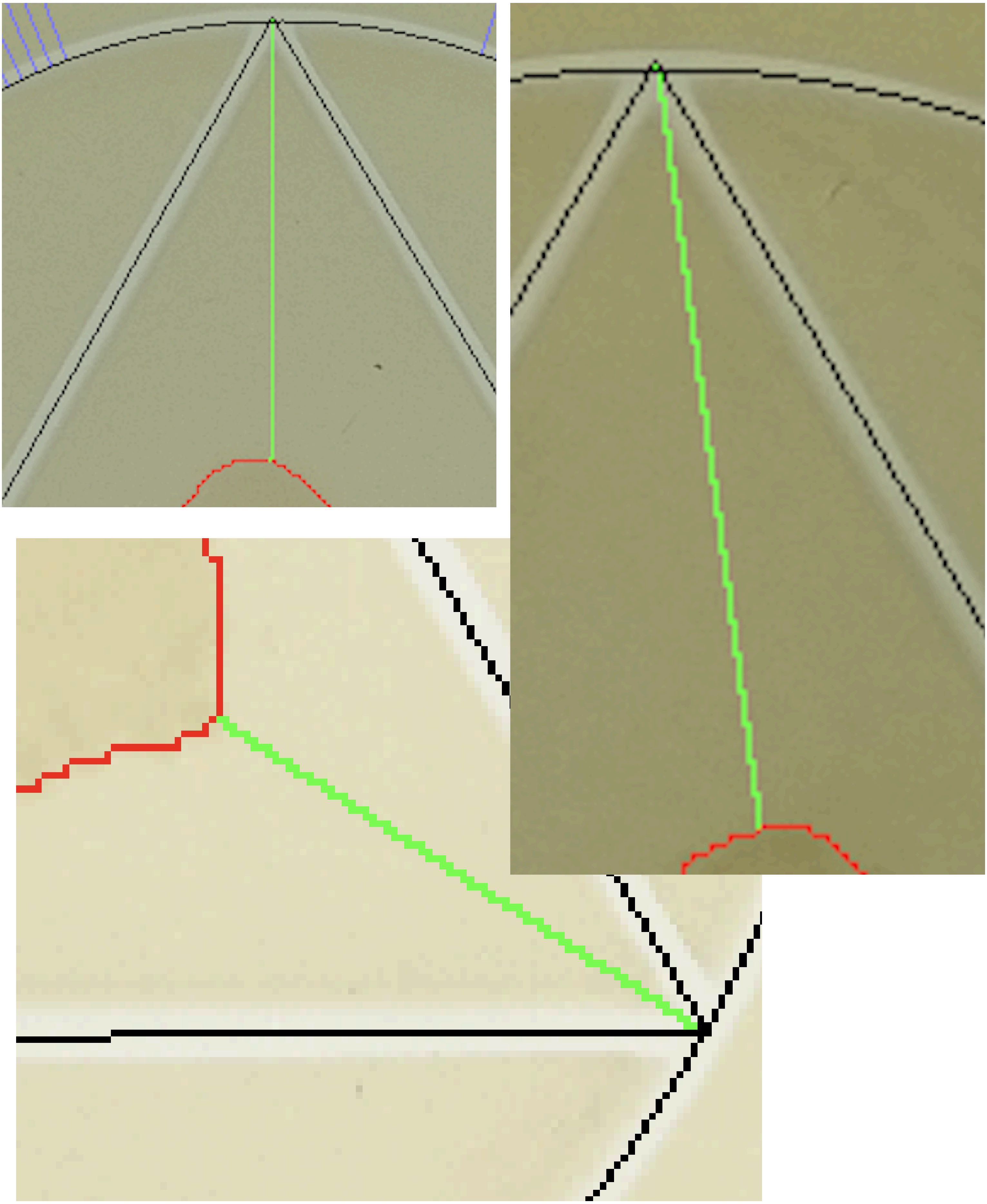

Weaknesses and Suggestions for The References section:

1. Inconsistent Citation Style:

- There are inconsistencies in formatting, such as missing capitalization of proper nouns (e.g., "nature" instead of "Nature") and inconsistent punctuation (e.g., missing periods after journal abbreviations).
  The particular misspelled reference was removed, and all citations items were revised for correctness and consistency.

- Recommendation: Revise the references to adhere to a consistent citation style, depending on the journal's requirements.

2. Over-reliance on General Sources:

- Some references, such as general reviews on deep learning [23], might be less directly relevant to the specific methods used in the study.
  The reference has been removed and replaced with more relevant references to the study.

- Recommendation: Prioritize references that directly relate to the methodologies or concepts applied in this study, such as segmentation models or antibiotic resistance testing.
  A few references were added discussing related works in the introduction section.

3. Limited Context for Certain Citations:
- References like [4] and [5], which discuss specific antibiotic combinations, are poorly integrated into the study’s methodology or results discussion.

- Recommendation: Ensure that these references are explicitly linked to the rationale or implications of the study to enhance coherence.
  These references are highly relevant since they demonstrate that antibiotic interaction patterns can vary extensively across isolates, which is partly the motivation for developing the CombiANT method to begin with.

4. Lack of Historical Context:

- The references focus heavily on recent advances but could benefit from citing foundational works in bacterial resistance and image processing.
  Some references have been added relating to foundational work in image processing.

- Recommendation: Include seminal works in both fields to provide historical context and highlight how the study builds upon existing knowledge.
  We have changed parts of the introduction to encompass this.

5. Absence of Validation-Specific Citations:

- While the study emphasizes validation of the developed pipeline, there are limited references to methodologies for validating deep learning models or image analysis systems.

  One possibility would be to validate the bacterial segmentation using the intersection over union (IoU) metric or similar; however, we are not sure if such metrics would provide any meaningful insights and might instead add unnecessary detail, shifting focus away from the main point. We do not believe this segmentation is particularly difficult. With diverse annotated data resembling real cases, it should be solvable in all settings.

- Recommendation: Incorporate references to established validation protocols or metrics to strengthen the methodological rigor.

Reviewer #2:

It is funning that CombiANT is be used to identify suitable or inappropriate antibiotic combinations to deal with multidrug-resistant bacteria. If there is a another set of validation data, the results will be more credible.
The dataset used for validation (the three users) was specifically produced for the study, and the images were not seen when training the segmentation model. We would like to limit the scope of the study to the included validation data. As of the advances in deep learning and image processing in the 2020s, we don't believe that this image processing problem is particularly hard and that performance will extend to data captured in research or clinical setting, provided that the erroneous segmentations are annotated and the models retrained (which one would presume if this were to be implemented in a real setting).

Reviewer #3:

This is a useful example of a deep learning digital tool and improves on the current method of quantifying antibiotic interactions. Please add some text on how results were validated, and agreement between 3 reviewers was assessed.
Table 1 was added in the Results section with quantitative performance metric (mean absolute error).

Best regards,

Erik Hallström and the co-authors
